# Long-term cardiovascular risk after severe exacerbation of COPD: a population-based cohort study

**DOI:** 10.1183/23120541.00939-2025

**Published:** 2026-03-16

**Authors:** Yinqi Ding, Jingcen Hu, Canqing Yu, Dianjianyi Sun, Pei Pei, Ling Yang, Yiping Chen, Huaidong Du, Zengzhi Zhang, Maxim Barnard, Junshi Chen, Zhengming Chen, Liming Li, Jun Lv

**Affiliations:** 1 Department of Epidemiology & Biostatistics, School of Public Health, Peking University, Beijing, China; 2 Peking University Center for Public Health and Epidemic Preparedness & Response, Beijing, China; 3 Key Laboratory of Epidemiology of Major Diseases (Peking University), Ministry of Education, Beijing, China; 4 Clinical Trial Service Unit & Epidemiological Studies Unit (CTSU), Nuffield Department of Population Health, University of Oxford, UK; 5 Shinan Center for Disease Control and Prevention, Qingdao, China; 6 China National Center for Food Safety Risk Assessment, Beijing, China; 7 State Key Laboratory of Vascular Homeostasis and Remodeling, Peking University, Beijing, China; 8 The members of steering committee and collaborative group are listed in the supplementary material

## Abstract

**Background:**

Exacerbation of COPD (ECOPD) has been linked to increased cardiovascular disease (CVD) risk within the first year, yet longer term risk is unclear. We aimed to investigate the short-term and long-term CVD risks after severe ECOPD.

**Methods:**

Patients with self-reported or spirometry-detected COPD at baseline and patients with newly documented COPD during follow-up were included from the China Kadoorie Biobank. Multiple data sources were used to collect information on ECOPD hospitalisation and CVD incidence during follow-up. Time-dependent Cox regression models were used to estimate the hazard ratios and 95% confidence intervals for each risk period following ECOPD compared to the baseline period.

**Results:**

Of the 46 514 patients included, 48.2% had screen-detected COPD, 26.2% had self-reported COPD and 25.6% had newly documented COPD. During a median 11-year follow-up, 1185 acute myocardial infarction, 5778 other ischaemic heart disease, 1078 heart failure, 2390 pulmonary heart disease, 4989 ischaemic stroke and 1648 intracerebral haemorrhage cases occurred. Post-ECOPD risks of all outcomes were prominently elevated, with first-week hazard ratios (95% CI) of 8.60 (5.40–13.70), 6.68 (5.16–8.65), 10.98 (6.74–17.89), 24.76 (19.40–31.60), 3.11 (2.16–4.48) and 2.40 (1.27–4.54), respectively. The risks diminished thereafter but could persist for 6 years or longer. All three categories of patients with COPD faced increased risks of most outcomes, with patients with COPD at baseline bearing higher post-ECOPD risks of other ischaemic heart disease and pulmonary heart disease.

**Conclusion:**

CVD risks increased considerably after ECOPD, with risks of cardiac diseases and ischaemic stroke increased for 6 years or longer. Patients with screen-detected COPD had a similar burden of ECOPD and subsequent CVD to patients with doctor-diagnosed COPD.

## Shareable abstract

**After severe ECOPD, many CVD risks increase considerably and can persist for over 6 years. Screen-detected COPD carries a similar burden of ECOPD and subsequent CVD as doctor-diagnosed COPD, suggesting an opportunity for early detection and treatment.** https://bit.ly/3KkVQT2

## Introduction

Patients with COPD face a higher risk of cardiovascular disease (CVD), partly due to systemic inflammation [1]. Exacerbations of COPD (ECOPD) are critical events in COPD progression, elevating the risk of hospitalisation, readmission and death [2]. Triggered by infections or irritants, ECOPD is characterised by acute surges in airway and systemic inflammation. The slow recovery of inflammatory biomarkers after ECOPD [3] implies a greater CVD risk compared to stable periods. CVD is a major COPD comorbidity; therefore, clarifying the degree, duration and subtypes of increased CVD risk after ECOPD may shed light on disease burden and prevention windows, enabling more tailored care and improved prognosis.

Previous studies have investigated post-exacerbation risk of CVD in COPD, using either administrative databases [4–9] or data from randomised controlled trials [10–12]. Most studies included patients with COPD who met specific criteria, such as smoking history [5, 8] and forced expiratory volume in 1 s (FEV_1_) <70% predicted [10, 11], often representing patients with more severe disease. However, approximately 70% of patients with COPD worldwide remain undiagnosed [13], and these patients have rarely been studied, leaving their risks unknown. Although CVD risks within 1 year of ECOPD have been investigated [5–7, 12], long-term risks are less clear. Additionally, while composite CVD [11, 12], stroke and myocardial infarction (MI) [5, 6] have been commonly examined, risk patterns for other CVD subtypes remain uncertain.

This study used data from the China Kadoorie Biobank (CKB), a prospective cohort, to investigate the short-term and long-term risks of various CVD subtypes following ECOPD through two analytic approaches. Associations were compared between patients with doctor-diagnosed and screen-detected COPD, with the latter more likely to be asymptomatic or mildly symptomatic.

## Methods

### Study design and participants

The CKB is a prospective cohort that recruited 512 723 participants aged 30–79 years from five rural and five urban purposively chosen regions in China during 2004–2008, gathering information through electronic questionnaires, physical examinations and blood sample collection. Participants have then been followed longitudinally for morbidity, mortality and hospitalisation by linking to the local disease and death registry systems and the national health insurance (HI) database, supplemented by active follow-up for those not linked to the HI database. All causes of disease and death were coded using the International Classification of Diseases 10th revision (ICD-10). Further details on CKB are available elsewhere [14]. All participants provided written informed consent. Approval was obtained by the Ethical Review Committee of the Chinese Center for Disease Control and Prevention (Beijing, China), the Peking University Institutional Review Board (Beijing, China) and the Oxford Tropical Research Ethics Committee, University of Oxford (UK).

**FIGURE 1:**
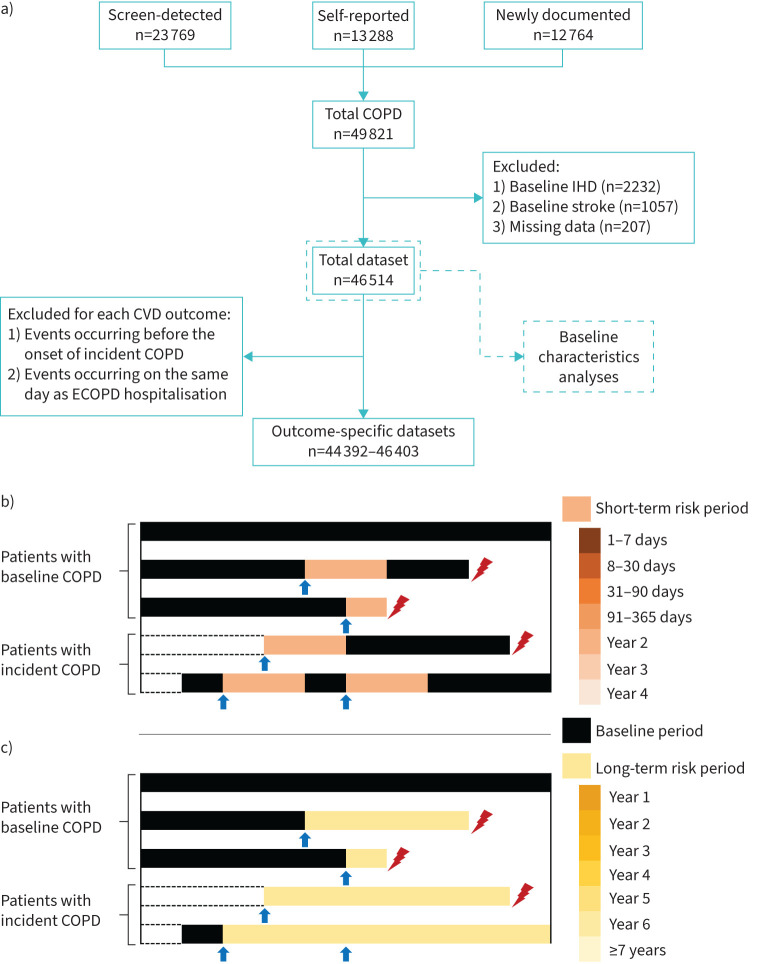
a) Flow chart of participants. b, c) Study design and two analytical strategies adopted in this study. Each strip represents a participant, including patients with baseline (screen-detected or self-reported) COPD and newly documented (incident) COPD during follow-up, with the latter starting the observation period at the moment of their first COPD occurrence during follow-up. The blue arrows indicate an exacerbation of COPD (ECOPD) hospitalisation, while the red lightning represents the first cardiovascular disease (CVD) event. The black part of the strip represents the baseline period, while the colour part represents the risk period. The short-term risk period was defined as 1–7 days, 8–30 days, 31–90 days, 91–365 days, year 2, year 3 and year 4 following each ECOPD hospitalisation. The long-term risk period was defined as year 1, year 2, year 3, year 4, year 5, year 6 and ≥7 years following the first ECOPD hospitalisation during follow-up. IHD: ischaemic heart disease.

This study categorised patients into three types depending on their COPD diagnosis (figure 1): 1) self-reported: reporting a prior doctor-diagnosed emphysema or bronchitis in the baseline questionnaire survey (n=13 288); 2) screen-detected: no prior report but baseline spirometry meeting the fixed ratio criterion (FEV_1_/forced vital capacity (FVC) <0.7) (n=23 769); and 3) newly documented (incident): not in the above two categories but diagnosed (ICD-10, J41–J44 in primary diagnosis) during follow-up (n=12 764).

### Ascertainment of ECOPD hospitalisation

This study focused on severe ECOPD events requiring hospitalisation, as defined by the Global Initiative for Chronic Obstructive Lung Disease (GOLD) [2]. We used the ICD-10 codes J41–J44 in the primary diagnosis to capture all ECOPD hospitalisations from the HI database, as per other studies [15, 16]. We excluded hospitalisations lasting <1 day or >30 days or occurring within 14 days after a preceding one to avoid including repeated episodes in the same course [8].

### Ascertainment of CVD outcomes

CVD outcomes were identified from disease and death registries, HI databases and annual active follow-ups. These included acute myocardial infarction (AMI), other ischaemic heart disease (IHD), heart failure, pulmonary heart disease, ischaemic stroke (IS) and intracerebral haemorrhage (ICH). The specific ICD-10 codes for these outcomes are detailed in table S1. CKB is currently conducting independent clinical adjudication on retrieved medical records for CVD cases.

### Assessment of covariates

The baseline questionnaire collected data on sociodemographic characteristics (age, sex, education, occupation, income, marital status), lifestyle habits (smoking, alcohol consumption, diet, physical activity), household air pollution, passive smoking, and personal and family medical histories. Trained staff measured height, weight, waist circumference, blood pressure and blood glucose following a standard protocol, and body mass index was calculated.

### Statistical analysis

From the total dataset, we excluded participants who 1) reported IHD (n=2232) or stroke (n=1057) at baseline or 2) lacked data on variables of interest (n=207) (figure 1a). For each CVD outcome, we further excluded 1) patients with incident COPD who developed outcome events before COPD onset and 2) patients with outcomes and ECOPD on the same day, owing to an unclear temporal sequence. Outcome-specific datasets for association analyses were created.

We reported participants' baseline characteristics in the total dataset, grouping by the timing of COPD onset or the number of ECOPD during the observation period. Continuous and categorical variables were reported as mean±sd and percentage, respectively, and p-values for group comparisons were calculated using ANOVA and the chi-square test accordingly.

With age as the time scale, the observation started at baseline for patients with COPD already diagnosed at baseline (prevalent COPD), and the time-to-first COPD occurrence during follow-up for incident COPD, and ended on the date of the CVD outcome of interest, death, loss to follow-up, or 31 December 2018, whichever occurred first (figure 1b, c). In the analyses of heart failure and pulmonary heart disease, participants who developed other CVD outcomes before these two were censored at the earliest incidence date of the other CVD outcomes [17].

We adopted two analytic strategies typically used in previous studies [8, 10–12]. The first examined short-term CVD risk following any ECOPD hospitalisation (figure 1b). Because 95% of inter-exacerbation intervals were ≤4 years (table S2), we set the risk period as day 1 to 4 years post-exacerbation, the next exacerbation, or the end of observation. This included specific intervals: 1–7 days, 8–30 days, 31–90 days, 91–365 days, year 2, year 3 and year 4. The baseline period was the remainder outside the risk period, and for those without ECOPD, the whole observation period. We further distinguished and analysed the “first” and “second and subsequent” ECOPD separately, using the same baseline reference. The second strategy estimated long-term CVD risk following the first ECOPD hospitalisation (figure 1c). The baseline period was the time before the first ECOPD, or the entire observation period for ECOPD-free patients, while the risk period was all time after the first ECOPD, classified into year 1, year 2, year 3, year 4, year 5, year 6 and ≥7 years.

We used time-dependent Cox regression models to estimate hazard ratios (HRs) and 95% confidence intervals (CIs) for risk periods *versus* baseline periods, stratified by 10-year age groups at each period's beginning and 10 survey areas. We adjusted for the majority of potential shared risk factors between COPD and CVD based on previous reviews and studies [8, 18, 19], including baseline age; sex; education; occupation; marital status; household income; smoking; alcohol consumption; physical activity; intake frequencies of red meat, fruits and vegetables; body mass index; waist circumference; cooking and heating fuel types; years of solid fuel use; stove ventilation; second-hand smoke exposure; FEV_1_ % predicted; family histories of heart disease and stroke; COPD treatment; CVD-related medication use; asthma; and hypertension and diabetes at baseline or follow-up.

Sensitivity analyses included 1) identifying ECOPD using J44.0–J44.1 in the primary diagnosis [20, 21]; 2) defining an ECOPD course as 28 days to ensure the robustness of the results irrespective of the definition used; 3) defining screen-detected COPD using the lower limit of normal criterion (FEV_1_/FVC lower than the bottom 5% of the healthy population's normal distribution) [2]; 4) employing the Fine and Gray model to account for competing risks; and 5) excluding patients with two or more ECOPDs from long-term risk analyses to avoid the impact of subsequent events on the first's results.

Subgroup analyses were stratified by age, sex, region, smoking status and COPD type, with interaction terms evaluated using likelihood ratio tests. To increase statistical power, risk periods with similar effects were combined: short-term into 1–30 days, 31–365 days, year 2 and years 3–4; and long-term into years 1–2, years 3–4, years 5–6 and ≥7 years.

All analyses applied Bonferroni correction for multiple comparisons, setting the significance threshold as 0.05 divided by the number of tests within each analysis. Stata 17.0 (StataCorp, TX, USA) and R 4.3.1 (www.r-project.org) were used to perform all the analyses. Further details on methods can be found in the supplementary methods.

## Results

### Baseline characteristics of study participants

The study included 46 514 participants, with a mean baseline age of 58.6±10.4 years, 49.6% men and 66.9% living in rural regions. During follow-up, 21.1% of participants experienced ECOPD once, and 15.9% at least twice (table 1). Patients with multiple ECOPD were more often men, rural residents and ever-smokers, with a lower FEV_1_ % predicted and a higher prevalence of respiratory symptoms at baseline. Patients with screen-detected, self-reported and newly documented COPD accounted for 48.2%, 26.2% and 25.6% of the total, respectively. The ages at baseline and the first ECOPD occurrence were comparable across groups. Patients with self-reported COPD were less likely to be ever-smokers, weekly drinkers or exposed to solid fuels, but they exhibited the most severe airflow obstruction, symptoms and exacerbation frequency (table S2). Patients with screen-detected COPD showed moderate impairment, whereas patients with newly documented COPD had the best spirometry results and fewest symptoms.

**TABLE 1:** Baseline characteristics of study participants

	Based on the number of ECOPD			Based on the timing of COPD onset		
	None	1	≥2	Screen-detected	Self-reported	Newly documented
**Participants (n)**	29 277	9828	7409	22 417	12 176	11 921
**Age at baseline (years)**	57.1±10.9	60.0±9.5	62.3±8.3	58.3±10.9	57.8±10.4	59.8±9.4
**Age at the start of observation (years)**	57.3±11.0	66.1±9.8	65.7±8.9	58.3±10.9	57.8±10.4	67.3±9.7
**Age at the first ECOPD occurrence**^#^ **(years)**	-	68.3±9.6	68.9±8.5	70.2±8.5	68.5±8.4	68.0±9.5
**Male sex** ^¶^	48.1	49.6	55.5	49.8	49.4	49.4
**Rural location**	61.3	73.7	80.2	68.0	55.6	76.4
**Education below middle school**	66.5	73.8	81.4	71.3	64.3	74.8
**Industrial and agricultural workers**	55.8	61.0	66.3	61.5	49.5	62.4
**Annual household income <20 000 CNY**	63.7	69.1	74.0	71.2	58.0	66.5
**Married**	86.6	85.2	83.1	84.8	87.6	85.7
**Ever-smoker among**						
Women	6.3	13.0	22.7	9.9	7.4	13.0
Men	78.3	82.6	85.5	81.5	75.7	83.3
**Currently weekly drinker** ^+^	16.8	17.7	18.0	18.6	13.8	17.9
**Physical activity (MET-h·day^−1^)**	19.8±14.1	17.8±12.3	16.6±11.8	19.4±13.9	18.3±13.5	18.3±12.6
**Regular consumption of**						
Red meat	23.2	22.4	20.4	21.0	25.8	22.3
Fresh vegetables^§^	95.0	95.4	96.1	95.6	94.9	95.0
Fresh fruits	14.3	10.8	8.5	11.6	16.9	10.2
**Body mass index (kg·m^−2^)**	22.9±3.4	22.8±3.6	21.9±3.7	22.5±3.3	23.0±3.8	22.9±3.6
**Waist circumference (cm)**	78.6±10.0	79.0±10.3	77.3±10.7	77.6±9.7	79.5±10.6	79.2±10.3
**Current cooking with solid fuels**	39.8	47.3	51.2	44.4	36.0	48.2
**Stove ventilation in the baseline house**	72.4	74.7	72.5	68.2	78.5	76.1
**Current heating with solid fuels**	30.6	35.2	33.5	32.0	27.4	36.7
**Time lived with smokers (years)**	24.1±20.1	27.0±21.5	28.6±22.5	25.0±20.9	25.4±20.3	26.3±21.4
**Family history of**						
Heart attack	2.8	2.2	1.8	2.4	3.1	2.1
Stroke	16.6	14.4	12.0	14.9	18.1	13.6
**FEV_1_ % predicted**	71.9±21.2	76.3±22.9	61.4±23.6	66.0±19.2	70.7±26.8	81.2±19.7
**Respiratory symptoms** ^ƒ^	30.9	29.1	43.4	24.3	55.9	23.9
**Receiving treatment for COPD**	8.4	5.4	13.5	-	32.8	-
**Using drugs for CVD** ^##,^^¶¶^	4.4	5.1	4.6	4.2	4.7	5.1
**Asthma at baseline**	2.6	2.3	4.1	1.8	5.7	1.5
**Comorbidities at baseline or follow-up**						
Hypertension	44.4	48.6	49.9	44.9	44.1	50.5
Diabetes	10.3	12.0	13.6	9.9	11.5	13.3

Data are presented as mean±sd or %. All p-values were <0.001 unless otherwise indicated. CNY: Chinese Yuan; CVD: cardiovascular disease; ECOPD: exacerbation of COPD; FEV_1_: forced expiratory volume in 1 s; MET: metabolic equivalent of task. ^#^: only analysed for participants with ≥1 ECOPD hospitalisation; ^¶^: p=0.615 based on timing of COPD onset; ^+^: p=0.015 based on the number of ECOPD; ^§^: p=0.004 based on the timing of COPD onset; ^ƒ^: include shortness of breath while walking, slowing down while walking due to chest discomfort, coughing frequently last year and coughing up sputum frequently last year; ^##^: include aspirin, angiotensin-converting enzyme inhibitors, β-blockers, statins, diuretics and calcium antagonists; ^¶¶^: p=0.018 based on the number of ECOPD.

### Associations between ECOPD hospitalisation and short-term CVD risk

Sample sizes across outcome-specific datasets ranged from 44 392 to 46 403, with a median follow-up of 11 years. Events comprised 1185 AMI, 5778 other IHD, 1078 heart failure, 2390 pulmonary heart disease, 4989 IS and 1648 ICH (table S3).

**FIGURE 2:**
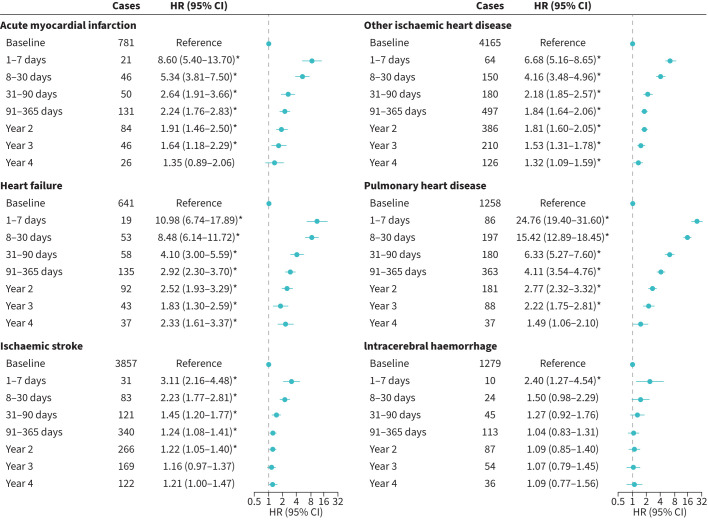
Associations between hospitalisation for exacerbation of COPD (ECOPD) and short-term cardiovascular disease (CVD) risk. The multivariable models were adjusted for the same covariates as in table 2. HR: hazard ratio. *: p<0.05/6 after Bonferroni correction.

After adjusting for covariates, we observed heightened risks of all CVD outcomes following ECOPD hospitalisation, peaking within the first week, with HRs (95% CIs) of 8.60 (5.40–13.70) for AMI, 6.68 (5.16–8.65) for other IHD, 10.98 (6.74–17.89) for heart failure, 24.76 (19.40–31.60) for pulmonary heart disease, 3.11 (2.16–4.48) for IS and 2.40 (1.27–4.54) for ICH (figure 2). These risks fell over time but remained elevated for AMI, other IHD, heart failure and pulmonary heart disease until the year 3 or 4 after multiple corrections. However, the cerebrovascular risks were short-lasting: 2 years for IS and 1 week for ICH. Both first and second and subsequent episodes showed similar association trends (table 2). However, the association between the second and subsequent episodes and pulmonary heart disease was constantly stronger than with that of the first within 3 years.

**TABLE 2:** Associations between first or subsequent ECOPD hospitalisation and short-term CVD risk

	First ECOPD		Second and subsequent ECOPD	
	Cases (n)	HR (95% CI)	Cases	HR (95% CI)
**Acute myocardial infarction**				
Baseline	781	Reference	781	Reference
1–7 days	6	6.85 (2.99–15.67)*	15	9.23 (5.31–16.07)*
8–30 days	17	5.66 (3.40–9.43)*	29	4.80 (3.14–7.32)*
31–90 days	18	2.51 (1.53–4.11)*	32	2.54 (1.70–3.80)*
91–365 days	63	2.27 (1.69–3.05)*	68	2.06 (1.51–2.82)*
Year 2	51	1.92 (1.39–2.64)*	33	1.79 (1.21–2.66)*
Year 3	36	1.82 (1.26–2.64)*	10	1.09 (0.57–2.09)
Year 4	19	1.26 (0.78–2.05)	7	1.54 (0.72–3.33)
**Other ischaemic heart disease**				
Baseline	4165	Reference	4165	Reference
1–7 days	29	7.05 (4.84–10.28)*	35	5.85 (4.13–8.28)*
8–30 days	60	3.76 (2.89–4.91)*	90	4.11 (3.27–5.16)*
31–90 days	76	1.97 (1.55–2.49)*	104	2.19 (1.77–2.71)*
91–365 days	251	1.66 (1.44–1.91)*	246	1.88 (1.62–2.19)*
Year 2	260	1.78 (1.55–2.06)*	126	1.67 (1.37–2.03)*
Year 3	143	1.36 (1.14–1.63)*	67	1.73 (1.34–2.24)*
Year 4	98	1.23 (0.99–1.52)	28	1.39 (0.95–2.04)
**Heart failure**				
Baseline	641	Reference	641	Reference
1–7 days	7	11.20 (5.19–24.20)*	12	9.57 (5.17–17.72)*
8–30 days	18	6.76 (4.11–11.12)*	35	7.78 (5.22–11.61)*
31–90 days	22	3.40 (2.16–5.34)*	36	3.97 (2.69–5.85)*
91–365 days	59	2.26 (1.67–3.07)*	76	2.98 (2.20–4.05)*
Year 2	59	2.38 (1.75–3.24)*	33	2.10 (1.41–3.13)*
Year 3	29	1.57 (1.05–2.36)	14	1.80 (1.02–3.17)
Year 4	30	2.16 (1.44–3.24)*	7	1.71 (0.79–3.71)
**Pulmonary heart disease**				
Baseline	1258	Reference	1258	Reference
1–7 days	30	20.37 (13.69–30.31)*	56	26.40 (19.48–35.80)*
8–30 days	57	11.61 (8.71–15.48)*	140	18.12 (14.57–22.52)*
31–90 days	60	4.90 (3.71–6.47)*	120	7.24 (5.77–9.07)*
91–365 days	142	3.09 (2.54–3.76)*	221	4.96 (4.11–5.99)*
Year 2	96	2.34 (1.86–2.94)*	85	3.39 (2.63–4.38)*
Year 3	57	2.08 (1.57–2.77)*	31	2.43 (1.65–3.56)*
Year 4	28	1.52 (1.03–2.25)	9	1.41 (0.72–2.77)
**Ischaemic stroke**				
Baseline	3857	Reference	3857	Reference
1–7 days	9	2.36 (1.22–4.58)	22	3.30 (2.13–5.12)*
8–30 days	35	2.41 (1.71–3.39)*	48	2.01 (1.49–2.73)*
31–90 days	55	1.49 (1.13–1.97)	66	1.34 (1.03–1.74)
91–365 days	198	1.35 (1.16–1.59)*	142	1.03 (0.85–1.25)
Year 2	177	1.23 (1.05–1.46)	89	1.12 (0.89–1.40)
Year 3	120	1.13 (0.93–1.37)	49	1.16 (0.86–1.56)
Year 4	95	1.18 (0.95–1.46)	27	1.19 (0.80–1.76)
**Intracerebral haemorrhage**				
Baseline	1279	Reference	1279	Reference
1–7 days	7	4.82 (2.25–10.33)*	3	0.99 (0.31–3.13)
8–30 days	7	1.30 (0.61–2.75)	17	1.53 (0.92–2.55)
31–90 days	13	0.98 (0.56–1.72)	32	1.36 (0.92–2.01)
91–365 days	57	1.11 (0.83–1.48)	56	0.93 (0.68–1.26)
Year 2	53	1.10 (0.82–1.49)	34	1.04 (0.72–1.52)
Year 3	32	0.94 (0.64–1.36)	22	1.38 (0.88–2.17)
Year 4	28	1.12 (0.75–1.67)	8	0.99 (0.48–2.02)

Multivariable models were adjusted for age; sex; level of education; occupation; marital status; household income; tobacco smoking; alcohol consumption; physical activity; intake frequencies of red meat, fruits and vegetables; body mass index; waist circumference; fuel types currently used in cooking or heating; years of cooking or heating with solid fuels; stove ventilation in the baseline house; exposure status to second-hand smoke; forced expiratory volume in 1 s % predicted; family history of heart disease and stroke; baseline asthma; COPD treatment and the use of CVD-related medications; and hypertension and diabetes discovered at baseline or follow-up (time-dependent). CVD: cardiovascular disease; ECOPD: exacerbation of COPD; HR: hazard ratio. *: p<0.05/12 after Bonferroni correction.

### Associations between first ECOPD hospitalisation and long-term CVD risk

**FIGURE 3:**
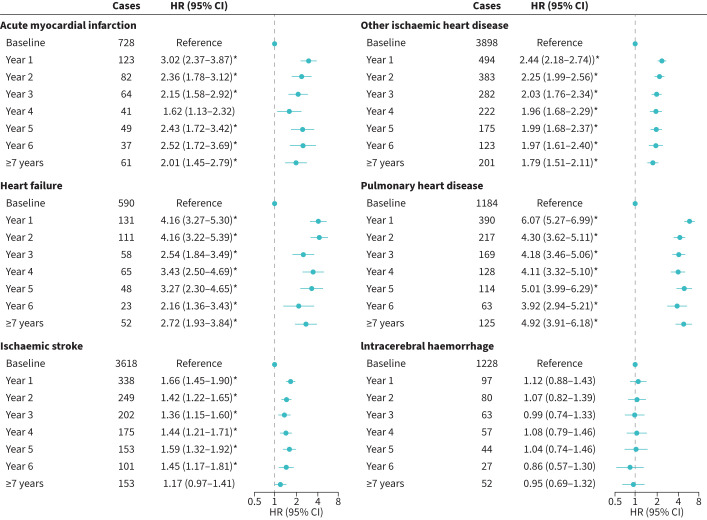
Associations between first hospitalisation for exacerbation of COPD (ECOPD) and long-term cardiovascular disease (CVD) risk. The multivariable models were adjusted for the same covariates as in table 2. HR: hazard ratio. *: p<0.05/6 after Bonferroni correction.

Following the first ECOPD, the risks of AMI, other IHD, heart failure and pulmonary heart disease increased persistently for over 6 years, with HRs (95% CIs) for ≥7 years of 2.01 (1.45–2.79), 1.79 (1.51–2.11), 2.72 (1.93–3.84) and 4.92 (3.91–6.18) (figure 3). IS risk remained elevated in year 6, with an HR (95% CI) of 1.45 (1.17–1.81). Analyses of patients with only one ECOPD during follow-up demonstrated stronger associations and clearer reduction trends over time (figure S1). The HRs (95% CIs) of ICH increased to 1.94 (1.49–2.53) in year 1 and 1.62 (1.18–2.22) in year 2, and risks of other outcomes were prolonged beyond year 6.

### Sensitivity analyses

The results of ECOPD identified by J44.0–J44.1 were generally similar to primary analyses, with wider confidence intervals reflecting reduced statistical power (tables S4 and S5). Altering the criteria for the ECOPD course and screen-detected COPD did not change the results materially. Accounting for competing risks, participants with exacerbations had greater cumulative incidences of most outcomes except ICH (figure S2). The subdistribution HRs were mostly consistent with the main findings, though slightly lower than the HRs (figures S3 and S4).

### Subgroup analyses

**FIGURE 4:**
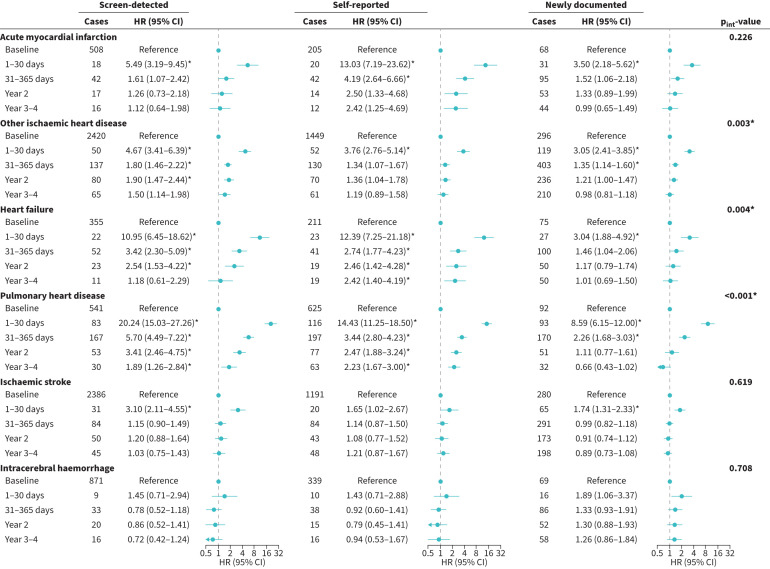
Associations between hospitalisation for exacerbation of COPD (ECOPD) and short-term cardiovascular disease (CVD) risk stratified by different types of COPD patients. The multivariable models were adjusted for the same covariates as in table 2. CI: confidence interval; HR: hazard ratio. *: p<0.05/18 after Bonferroni correction and p_int_<0.05/6.

Patients aged <60 years had higher post-ECOPD risks of other IHD and pulmonary heart disease than those aged ≥60 years (tables S6 and S7). There were no significant sex difference in CVD risks (tables S8 and S9). The risk of other IHD after ECOPD was more pronounced in rural than urban patients (tables S10 and S11). The associations between ECOPD and heart failure were greater in never-smokers than in ever-smokers (tables S12 and S13). Similar findings to the primary analyses were observed across the three COPD types, though risks were diminished (figure 4 and figure S5). Compared to patients with newly documented COPD, the other two groups had higher post-ECOPD risks of other IHD and pulmonary heart disease (p_int_=0.003 and p_int_<0.001 for short-term risk; both p_int_<0.001 for long-term risk).

## Discussion

In this study of Chinese patients with COPD, risks of AMI, other IHD, heart failure, pulmonary heart disease and IS spiked within 30 days after ECOPD hospitalisation and then decreased, but persisted for 6 years or longer. All groups of patients with COPD were at increased CVD risk, with patients having COPD at baseline bearing higher post-ECOPD risks of other IHD and pulmonary heart disease than patients with newly documented COPD.

Smoking is widely used for COPD diagnosis in many studies. A UK retrospective cohort of 213 466 smokers with COPD found that severe ECOPD increased CVD risks for over a year [8]. Another self-controlled case series study of ever-smokers reported elevated risks of MI and IS after ECOPD, which remained higher until 15–28 days [5]. Results and trends from that study were similar to ours in ever-smokers (table S12), despite differences in risk duration due to patient inclusion and study design. We also discovered consistent relationships among never-smokers, who had even higher post-ECOPD risk of heart failure than smokers, providing fresh insights into this understudied group. Nearly half of patients with COPD worldwide are non-smokers, especially in developing countries [22], and evidence of non-smoking risk factors has been increasingly recognised [23]. The findings for never-smokers merit further investigation in future research.

Another retrospective cohort study included 14 713 patients with COPD regardless of smoking status with a median follow-up of 41.3 months, drawn from the Yinzhou electronic health records database in China [24]. The study reported that patients with a history of severe exacerbations had a higher incidence of heart failure decompensation, cerebral ischaemia and arrhythmias. Our findings corroborate and expand upon these results, benefiting from a larger sample, finer time division, stringent statistical models and comprehensive information on confounders.

We observed that cardiac diseases, specifically IHD, heart failure and pulmonary heart disease, showed higher relative risks after ECOPD than stroke. Most studies with similar findings only included total stroke or IS [5, 8, 10, 25]. We further found that the association of ECOPD with ICH was weaker than with IS. Another self-controlled case series study reported similar risks for incident or recurrent IS and haemorrhagic stroke [4]. Moreover, we evaluated risk over periods ranging from the first year up to 13.7 years, revealing that cardiac risk persisted longer than 6 years while cerebrovascular risk lasted less.

In our study, the relationships between ECOPD and CVD differed slightly across three COPD types, likely reflecting differences in patient characteristics. Patients with COPD newly documented during follow-up had shorter disease durations, better baseline spirometry and fewer symptoms, while the other two groups had apparently worse baseline airflow and symptoms and experienced more exacerbations thereafter. The similar respiratory and cardiovascular burden between patients with screen-detected and self-reported COPD is worth noting. A previous study also found that patients with physician-diagnosed and undiagnosed COPD had comparable healthcare service utilisation for ECOPD [26].

We observed sharply increased short-term CVD risks and relatively stable long-term risks after ECOPD. Chronic inflammation and CVD risk factors in COPD patients promote atherosclerosis [27] and vascular and myocardial remodelling [28] for years. ECOPD, by causing an acute spike in inflammation [29, 30], exacerbates these chronic changes and adds temporary CVD-promoting factors, resulting in a remarkable CVD risk in the short term [31]. Potentially prolonged and incomplete recovery and lifestyle changes after exacerbation further cumulate the CV risk factors and account for the longer term risk [31]. Early COPD identification and proactive exacerbation prevention may help slow and stop the deterioration spiral and lower cardiopulmonary risk [31].

To our knowledge, this is the first population-based prospective cohort study incorporating a field spirometry test, enabling the inclusion of patients with asymptomatic or mild, undiagnosed COPD and comparison of their CVD risk with symptomatic clinical patients. We had statistical power to examine the effect patterns of ECOPD on different CVD subtypes with the large number of CVD cases collected through multiple ways. The lengthy follow-up period allowed us to employ two analytic methods to address both short-term and long-term impact spanning more than 6 years.

This study has several limitations. First, the three groups of patients with COPD had distinct characteristics, but largely consistent findings across groups justified their combination in primary analysis. Second, capturing newly diagnosed COPD from healthcare-related data may have excluded patients with mildly symptomatic disease who did not seek care. Nonetheless, the patients identified during baseline spirometry tests compensated for this. Third, we focused only on severe events that required hospitalisation. However, moderate exacerbations are less reliably identified by using nonspecific COPD drugs as a proxy [7] and led to an apparently lower CVD risk [8]. Fourth, although misclassifying MI or heart failure as ECOPD was possible, validation work revealed good diagnostic accuracy for COPD and CVD [32, 33]. Strong associations, particularly for less confusable outcomes like IS and ICH, were unlikely to be explained by misclassification. Fifth, bronchodilators were not used during spirometry tests due to practical constraints in a large field survey. Sixth, self-reported variables may introduce nondifferential misclassifications, leading the results to the null hypothesis. Finally, while the non-representative nature of CKB does not preclude the internal validity of the study's findings, caution should be taken when applying these findings to other populations.

### Conclusion

This cohort study of Chinese patients with COPD revealed that after an exacerbation, the risk of all CVD subtypes increased remarkably. The risk declined but persisted 1 month later, and could last 6 years or more. There are a considerable number of patients with undiagnosed COPD globally [13], and many diagnosed patients have not been prescribed medication [34] or have poor adherence [35, 36], which may contribute to the high burden of exacerbation and associated CVD. More studies are needed to determine whether early detection and enhanced therapeutic management of COPD can reduce the related cardiovascular burden.

## Data Availability

The access policy and procedures are available at www.ckbiobank.org.

## Supplementary material

**Please note:** supplementary material is not edited by the Editorial Office, and is uploaded as it has been supplied by the author. Supplementary material 00939-2025.SUPPLEMENT
