# Supplementary material for: Long-term cardiovascular risk after severe exacerbation of COPD: a population-based cohort study
Source: ERJ Open Res. 2026 Mar 16;12(2):00939-2025. doi: 10.1183/23120541.00939-2025 (PMC12991000; doi:10.1183/23120541.00939-2025)
Supplement: Supplementary file 1 [file 00939-2025.SUPPLEMENT.pdf]

Supplemental Material

Supplemental material for the manuscript entitled ***Long-term cardiovascular risk after severe exacerbation of chronic obstructive pulmonary disease: a population-based cohort study***

Contents

Members of the China Kadoorie Biobank collaborative group ....

Supplementary methods ....

Study design and implementation of China Kadoorie Biobank ....

Spirometry test at baseline ....

Assessment of covariates ....

Case adjudication ....

Adjustment for multivariable models ....

References ....

Table S1. ICD-10 codes for CVD outcomes ....

Table S2. Characteristics of lung function and respiratory symptoms among different COPD patients ....

Table S3. Characteristics of outcome-specific datasets ....

Table S4. Sensitivity analyses of the associations between ECOPD hospitalization and short-term CVD risk ....

Table S5. Sensitivity analyses of the associations between the first ECOPD hospitalization and long-term CVD risk ....

Table S6. Associations between ECOPD hospitalization and short-term CVD risk stratified by age ....

Table S7. Associations between first ECOPD hospitalization and long-term CVD risk stratified by age ....

Table S8. Associations between ECOPD hospitalization and short-term CVD risk stratified by sex ....

Table S9. Associations between first ECOPD hospitalization and long-term CVD risk stratified by

sex ....
Table S10. Associations between ECOPD hospitalization and short-term CVD risk stratified by study region ....
Table S11. Associations between first ECOPD hospitalization and long-term CVD risk stratified by study region..
Table S12. Associations between ECOPD hospitalization and short-term CVD risk stratified by smoking status..
Table S13. Associations between first ECOPD hospitalization and long-term CVD risk stratified by smoking status ....
Figure S1. Associations between first ECOPD hospitalization and long-term CVD risk in patients with only one hospitalization during follow-up ....
Figure S2. Cumulative incidence of CVD outcomes by the number of ECOPD ....
Figure S3. Subdistribution hazard ratios and 95% confidence intervals for short-term CVD risk after ECOPD hospitalization ....
Figure S4. Subdistribution hazard ratios and 95% confidence intervals for long-term CVD risk after the first ECOPD hospitalization ....
Figure S5. Associations between first ECOPD hospitalization and long-term CVD risk stratified by different types of COPD patients..

Members of the China Kadoorie Biobank collaborative group

International Steering Committee: Junshi Chen, Zhengming Chen (PI), Robert Clarke, Rory Collins, Liming Li (PI), Jun Lv, Richard Peto, Robin Walters.

International Co-ordinating Centre, Oxford: Daniel Avery, Maxim Barnard, Derrick Bennett, Ruth Boxall, Ka Hung Chan, Yiping Chen, Zhengming Chen, Charlotte Clarke, Johnathan Clarke; Robert Clarke, Huaidong Du, Ahmed Edris Mohamed, Hannah Fry, Simon Gilbert, Pek Kei Im, Andri Iona, Maria Kakkoura, Christiana Kartsonaki, Kshitij Kolhe, Hubert Lam, Kuang Lin, James Liu, Mohsen Mazidi, Iona Millwood, Sam Morris, Qunhua Nie, Alfred Pozarickij, Maryam Rahmati, Paul Ryder, Dan Schmidt, Becky Stevens, Iain Turnbull, Robin Walters, Baihan Wang, Lin Wang, Neil Wright, Ling Yang, Xiaoming Yang, Pang Yao.

National Co-ordinating Centre, Beijing: Xiao Han, Can Hou, Qingmei Xia, Chao Liu, Jun Lv, Pei Pei, Dianjianyi Sun, Canqing Yu, Lang Pan.

10 Regional Co-ordinating Centres:
Qingdao CDC: Zengchang Pang, Ruqin Gao, Shanpeng Li, Haiping Duan, Shaojie Wang, Yongmei Liu, Ranran Du, Yajing Zang, Liang Cheng, Xiaocao Tian, Hua Zhang, Yaoming Zhai, Feng Ning, Xiaohui Sun, Feifei Li. **Licang CDC:** Silu Lv, Junzheng Wang, Wei Hou. **Heilongjiang Provincial CDC:** Wei Sun, Shichun Yan, Xiaoming Cui. **Nangang CDC:** Chi Wang, Zhenyuan Wu,Yanjie Li, Quan Kang. **Hainan Provincial CDC:** Huiming Luo, Tingting Ou. **Meilan CDC:** Xiangyang Zheng, Zhendong Guo, Shukuan Wu, Yilei Li, Huimei Li. **Jiangsu Provincial CDC:** Ming Wu, Yonglin Zhou, Jinyi Zhou, Ran Tao, Jie Yang, Jian Su. **Suzhou CDC:** Fang Liu, Jun Zhang, Yihe Hu, Yan Lu, Liangcai Ma, Aiyu Tang, Shuo Zhang, Jianrong Jin, Jingchao Liu. **Guangxi Provincial CDC:** Mei Lin, Zhenzhen Lu. **Liuzhou CDC:** Lifang Zhou, Changping Xie, Jian Lan,Tingping Zhu,Yun Liu, Liuping Wei, Liyuan Zhou, Ningyu Chen, Yulu Qin, Sisi Wang. **Sichuan Provincial CDC:** Xianping Wu, Ningmei Zhang, Xiaofang Chen, Xiaoyu Chang. **Pengzhou CDC:** Mingqiang Yuan, Xia Wu, Xiaofang Chen, Wei Jiang, Jiaqiu Liu, Qiang Sun. **Gansu Provincial CDC:** Faqing Chen, Xiaolan Ren, Caixia Dong. **Maiji CDC:** Hui Zhang, Enke Mao, Xiaoping Wang, Tao Wang, Xi zhang. **Henan Provincial CDC:** Kai Kang, Shixian Feng, Huizi Tian, Lei Fan. **Huixian CDC:** XiaoLin Li, Huarong Sun, Pan He, Xukui Zhang. **Zhejiang Provincial CDC:** Min Yu, Ruying Hu, Hao

Wang. **Tongxiang CDC**: Xiaoyi Zhang, Yuan Cao, Kaixu Xie, Lingli Chen, Dun Shen. **Hunan Provincial CDC:** Xiaojun Li, Donghui Jin, Li Yin, Huilin Liu, Zhongxi Fu. **Liuyang CDC:** Xin Xu, Hao Zhang, Jianwei Chen,Yuan Peng, Libo Zhang, Chan Qu.

Event Adjudication Clinicians:

Beijing Tiantan Hospital,Capital Medical University Shuya Li, Haiqiang Qin, Yongjun Wang, **Peking University People's Hospital** Qiling Chen, Jihua Wang, **The 1st Affiliated Hospital of Harbin Medical University** Xiaojia Sun, Lei Wang, Xun Wang, Liming Zhang, Shanshan Zhou, **The 2nd Affiliated Hospital of Harbin Medical University** Hongyuan Chen, Li Chen, Haiyan Gou, Weizhi Wang, Yanmei Zhu, Yulan Zhu, **The 2nd Hospital of Hebei Medical University** Ning Zhang, **Huashan Hospital** Xin Cheng, Qiang Dong, Yi Dong, Kun Fang, Yiting Mao, **Jinling Hospital** Yu An, Peiling Chen, Yinghua Chen, Zhihong Liu, Lihua Zhang **The People's Hospital of Liaoning Province** Xiaohong Chen, Naixin Jv, Xiaojiu Li, Liyang Liu, Yun Lu, Xiaona Xing, **Qingdao Fuwai Cardiovascular Hospital** Shihao You, **Shengjing Hospital of China Medical University** Xiaoli Cheng, Chaojun Gua, Jinping Jiang, Jingyi Liu, Shumei Ma, **Shenyang Military General Hospital** Xuefeng Yang, **The First People's Hospital of Shenyang** Xiaomo Du, Jian Xu, Xuecheng Yang, Xiaodi Zhao, **West China Hospital, Sichuan University** Zilong Hao, Ming Liu, Deren Wang, **The Second Affiliated Hospital of Suzhou University** Xiaoting Li, **Suzhou Kowloon Hospital Shanghai Jiao Tong University School of Medicine** Lili Hui, Zhanling Liao, Feng Liu, **Qingdao Fuwai Cardiovascular Hospital** Chunning Feng, Dejiang Ji, Fengxia Qu, Wenwen Yuan, **The First Affiliated Hospital of Zhengzhou University** Xin Fu, **Zhongshan Hospital,** Jing Ding, Peng Du, Lirong Jin, Yueshi Mao, Xin Wang.

Supplementary methods

Study design and implementation of the China Kadoorie Biobank

The China Kadoorie Biobank (CKB) study was launched between 2004 and 2008, with five urban and five rural areas purposively selected across China based on local disease patterns, exposure to certain risk factors, demographic stability, quality of death and disease registries, and local commitment and capacity [1]. All nondisabled permanent residents aged 35 to 74 in each region were invited. To encourage participation, individuals who were slightly outside the target age range were not turned down from taking part in the survey.

A total of 512,891 participants aged 30-79 were recruited, with response rates of approximately one-third (33% in rural areas and 27% in urban areas). After registering and providing written informed consent, participants underwent baseline assessments, including a laptop-based questionnaire, physical measurements, and blood sample collection. The interviewer-administered questionnaire with built-in quality control functions asked about demographic characteristics, socioeconomic status, lifestyle factors (diet, smoking, alcohol consumption, tea consumption, etc.), exposure to air pollution, medical history and medication use, physical activity, sleep and mental health, and reproductive history for women. Physical examinations were conducted using standard instruments calibrated at regular intervals and following an established protocol to measure height, weight, waist and hip circumferences, bioimpedance, lung function, carbon monoxide (CO), blood pressure, and heart rate. Random blood glucose and hepatitis B surface antigen were measured using spot blood tests. In addition, 10 ml of fasting blood samples were collected from each participant, and the time of their last meal was recorded. Throughout the survey, regular central monitoring was carried out to assess recruitment rates, the distribution of key variables, blood processing delays, and data consistency for both general and individual staff.

After the baseline survey was completed, participants' unique national identity was used to link them to the local disease and death registry systems and the national health insurance (HI) database for long-term follow-up of their incidence and mortality status. Nearly 97% of the cohort members have been linked to the HI database. For those who have not been linked, an active follow-up was undertaken annually to ascertain their hospitalization, death, and migration out of the survey area. By the end of 2018, the follow-up loss rate was 0.79%.

Spirometry test at baseline

At baseline, pre-bronchodilator forced expiratory volume in one second (FEV1) and forced vital capacity (FVC) were measured by trained staff using a handheld Micro Spirometer following recommended procedures [2, 3]. Two acceptable maneuvers were recorded per participant, with the highest values used for analysis. A quality-control survey involving 15,728 randomly selected participants was conducted 1–2 weeks after baseline, which demonstrated excellent reproducibility for both FEV1 ($r_{Spearman}$=0.913) and FVC ($r_{Spearman}$=0.912) between the two surveys.

Assessment of covariates

At baseline, participants were interviewed and classified as never smokers, occasional smokers, former smokers, and current smokers, with the first two combined in this study as never-smokers and the latter two as ever-smokers. Former or current smokers were then asked about their frequency, type, and amount of smoking, and quitters were additionally asked about their reasons for quitting. To avoid reverse causation, we assigned those who ceased smoking owing to illness to the current smoker group. Similarly, participants were also asked about the frequency, type, and quantity of present or previous alcohol intake, and the total grams of alcohol consumed in a typical day were calculated.

Physical activity data collected included the type of job, commuting, leisure exercise, and household chores that participants mainly performed last year, as well as the frequency and duration of those activities. The level of each type of activity was then multiplied by the number of hours to obtain the total physical activity (metabolic equivalent [MET] hours/day). We also asked participants how often they had eaten meat, fresh fruits, and fresh vegetables over the past 12 months, and included the median (7, 5, 2, 0.5, and 0 days per week, respectively) for each group (daily, 4-6 days per week, 1-3 days per week, monthly, and never/ rarely) in the model as a continuous variable. Height, weight, and waist circumference were measured by professionally trained staff using an optimized height meter, Tanita body fat measuring device, and a soft leather ruler, all following a standardized survey manual. Body mass index (kg/m^2) was calculated as weight (kg) divided by the square of height (m).

Participants were asked how long (in years) they had lived in their previous three homes, as well as how often they cooked, and what kind of fuel they used in each. Coal and wood were categorized as solid fuels, whereas gas and electricity were categorized as clean fuels. Those who cooked at least once a month were considered to cook regularly, and the total number of hours they cooked with solid fuels in the three houses was calculated. Participants were also asked whether they heated and what fuel they used in the three households, with central heating also classified as a clean fuel, and the total number of hours spent heating with solid fuels was computed. The ventilation habit was only asked for cooking, as heating is usually not vented to keep warm. We also asked participants how frequently they were exposed to other people's tobacco smoke, either at home, workplace, or in public settings for at least five minutes each time. Those who were exposed at least once a week were further asked about the number of days and hours they were exposed per week, as well as how long they had lived with a smoker, if applicable.

Participants were questioned about diagnoses of ischemic heart disease, stroke, and hypertension. Those confirming these conditions were then asked about their use of medications including aspirin, angiotensin-converting enzyme inhibitors, beta-blockers, statins, diuretics, and calcium antagonists. Data on heart attack and stroke in their parents and siblings was also gathered.

After a minimum five-minute seated rest, participants' blood pressure was measured twice by trained staff with a digital sphygmomanometer (Omron UA-779; Live Source) at baseline [4]. A third measurement was taken if the first two readings differed by >10 mmHg, with the last two readings recorded. Hypertension was defined as a mean systolic blood pressure ≥140 mmHg or diastolic ≥90 mmHg. Those with a physician's diagnosis of hypertension or on antihypertensive medication were also classified as hypertensive. Furthermore, follow-up diagnoses with ICD-10 codes I10-I12, O13, H35.0, I15.0, I15.1, I15.9, and I67.4 were considered hypertension cases.

At baseline, participants were asked if a doctor had diagnosed them with diabetes and underwent on-site random blood glucose testing, noting the time since their last meal [5]. Those who denied a diagnosis and had a random plasma glucose level between 7.8~11.0 mmol/L were asked to return for fasting blood glucose testing the following day. Diabetes was diagnosed if participants answered "yes" to the initial question or had a random plasma glucose of ≥7.0 mmol/L after an 8-hour fast or ≥11.1

mmol/L if the fast was less than 8 hours, or a fasting plasma glucose of ≥7.0 mmol/L on retesting. Long-term follow-up cases of diabetes were identified using ICD-10 code E10-E14.

Please refer to the study website (https://www.ckbiobank.org/study-resources/survey-data) for access to the study questionnaire.

Case adjudication

To ascertain the accuracy of the outcomes collected, China Kadoorie Biobank (CKB) has devised detailed procedures and bespoke IT systems to validate and adjudicate primary disease outcomes. Staff retrieved relevant information about cases from medical records, including discharge summaries, diagnostic test results, and medication use. Then, qualified and specialized doctors from various major Chinese hospitals were invited to adjudicate the diagnosis of these diseases based on the extensive diagnostic and therapeutic information retrieved.

As of 31 December 2018, 30,974 new cases of ischemic heart disease (IHD) during follow-up have been retrieved and adjudicated, with a diagnostic accuracy of 93.3%. So did the 39,319 medical records of strokes. According to the most recent International Classification of Diseases (ICD) 11th edition, when asymptomatic cerebral infarction was considered IS, the diagnostic accuracy of total stroke and the three subtypes, including ischaemic stroke (IS), subarachnoid haemorrhage (SAH), and intracerebral haemorrhage (ICH), was 96.3%, 91.2%, 94.5, and 92.5%, respectively [6].

Adjustment for multivariable models

Multivariable models were adjusted for baseline covariates including age (years), sex (men or women), educational level (below middle school, middle or high school, or college or higher), occupation (farmers, factory workers, unemployed, or others), marital status (married, widowed, separated or divorced, or never married), annual household income (<￥10,000, ￥10,000-￥19,999, or ≥￥20,000), tobacco smoking (never, former, daily smoking 1-14, 15-24, or ≥25 cigarettes or equivalent per day), alcohol consumption (less than weekly, former weekly, weekly but not daily, daily drinking <30, 30-59, or ≥60 g/day of pure alcohol), total physical activity (MET-hours per day), frequencies of intaking red meat, fruits, and vegetables (days per week),

body mass index (kg/m^2), waist circumference (cm), fuel types used for cooking (solid, clean, others, or not cooking regularly) and heating (solid, clean, others, or no winter heating), duration of using solid fuels for cooking (years) and heating (years), stove ventilation when cooking (all stoves ventilated, part of stoves ventilated, not ventilated, no cooking facilities), secondhand smoke exposure (<1day/week and <20y, <1day/week but ≥20y, ≥1day/week but <20y, ≥1day/week and ≥20y and <20h/week, or ≥1day/week and ≥20y and ≥20h/week), forced expiratory volume in one second percent predicted (%), family histories of heart disease and stroke (yes or no), baseline COPD treatment (yes or no), the use of CVD-related medications (yes or no), and the time-dependent status of hypertension and diabetes (yes or no).

References

1. Chen Z, Chen J, Collins R, Guo Y, Peto R, Wu F, Li L. China Kadoorie Biobank of 0.5 million people: survey methods, baseline characteristics and long-term follow-up. *Int J Epidemiol* 2011: 40(6): 1652-1666.

2. Standardization of Spirometry, 1994 Update. American Thoracic Society. *Am J Respir Crit Care Med* 1995: 152(3): 1107-1136.

3. Smith M, Li L, Augustyn M, Kurmi O, Chen J, Collins R, Guo Y, Han Y, Qin J, Xu G, Wang J, Bian Z, Zhou G, Peto R, Chen Z. Prevalence and correlates of airflow obstruction in ~317,000 never-smokers in China. *Eur Respir J* 2014: 44(1): 66-77.

4. Lewington S, Lacey B, Clarke R, Guo Y, Kong XL, Yang L, Chen Y, Bian Z, Chen J, Meng J, Xiong Y, He T, Pang Z, Zhang S, Collins R, Peto R, Li L, Chen Z. The Burden of Hypertension and Associated Risk for Cardiovascular Mortality in China. *JAMA Intern Med* 2016: 176(4): 524-532.

5. Bragg F, Holmes MV, Iona A, Guo Y, Du H, Chen Y, Bian Z, Yang L, Herrington W, Bennett D, Turnbull I, Liu Y, Feng S, Chen J, Clarke R, Collins R, Peto R, Li L, Chen Z. Association Between Diabetes and Cause-Specific Mortality in Rural and Urban Areas of China. *JAMA* 2017: 317(3): 280-289.

6. Turnbull I, Clarke R, Wright N, Guo Y, Kartsonaki C, Pei P, Hacker A, Yu C, Gilbert S, Yang L, Zhou J, Sansome S, Lv J, Li L, Chen Z, Chen Y. Diagnostic accuracy of major stroke types in Chinese adults: A clinical adjudication study involving 40,000 stroke cases. *Lancet Reg Health West Pac* 2022: 21: 100415.

Table S1. ICD-10 codes for CVD outcomes

Outcomes	ICD-10 codes
Acute myocardial infarction	I21
Other ischaemic heart disease	I20; I22-I25
Heart failure	I50
Pulmonary heart disease	I26-I27
Ischaemic stroke	I63
Intracerebral haemorrhage	I61

CVD, cardiovascular disease; ICD-10, International Classification of Diseases (ICD) 10th version.
All the codes listed were identified in primary diagnosis.

Table S2. Characteristics of lung function and respiratory symptoms among different COPD patients

	Baseline screen-detected			Baseline self-reported			Newly documented during follow-up		
	No ECOPD	1 ECOPD	≥2 ECOPD	No ECOPD	1 ECOPD	≥2 ECOPD	No ECOPD	1 ECOPD	≥2 ECOPD
No. of participants, n (proportion)	19,039 (84.9)	1,506 (6.7)	1,872 (8.4)	8,984 (73.8)	1,227 (10.1)	1,965 (16.1)	1,254 (10.5)	7,095 (59.5)	3,572 (30.0)
FEV1%P, mean (SD)	68.4 (18.5)	57.1 (18.3)	49.6 (16.7)	77.6 (24.8)	57.1 (23.9)	47.8 (20.8)	84.4 (18.3)	83.7 (19.0)	75.1 (20.2)
Having respiratory symptoms *, rate	21.6	35.6	42.6	52.0	64.1	68.4	19.8	21.6	30.0
No. of respiratory symptoms *, mean (SD)	0.4 (0.8)	0.7 (1.1)	0.9 (1.2)	1.0 (1.2)	1.5 (1.4)	1.7 (1.4)	0.3 (0.8)	0.4 (0.8)	0.6 (1.0)
Receiving treatment for COPD, rate	-	-	-	27.4	43.2	50.8	-	-	-
Using drugs for CVD †, rate	4.2	3.7	3.7	4.7	4.9	4.7	4.1	5.4	4.9

COPD, chronic obstructive pulmonary disease; ECOPD, exacerbation of COPD; FEV1%P, forced expiratory volume in 1 second (FEV1) percent predicted; SD, standard deviation.

* Respiratory symptoms include shortness of breath while walking, slowing down while walking due to chest discomfort, coughing frequently last year, and coughing up sputum frequently last year.

† Drugs for CVD include aspirin, angiotensin-converting enzyme inhibitors, beta-blockers, statins, diuretics, and calcium antagonists.

Table S3. Characteristics of outcome-specific datasets

	Acute myocardial infarction	Other Ischaemic heart disease	Heart failure	Pulmonary heart disease	Ischaemic stroke	Intracerebral haemorrhage
No. of participants	46,396	44,392	45,766	45,084	45,271	46,403
No. of cases	1,185	5,778	1,078	2,390	4,989	1,648
No. of participants with ≥1 ECOPD, n (%)	17,092 (36.8)	14,594 (32.9)	16,351 (35.7)	15,536 (34.5)	15,700 (34.7)	17,097 (36.8)
No. of participants with ≥2 ECOPD, n (%)	7,328 (15.8)	5,820 (13.1)	6,704 (14.6)	5,932 (13.2)	6,701 (14.8)	7,336 (15.8)
Total no. of ECOPD	41,681	32,166	37,278	31,540	38,147	41,800
Average no. of ECOPD	0.9 (2.2)	0.7 (1.8)	0.8 (2.0)	0.7 (1.6)	0.8 (2.1)	0.9 (2.2)
Median (IQR) interval [*], year	0.5 (0.2-1.2)	0.6 (0.2-1.3)	0.6 (0.2-1.3)	0.6 (0.2-1.5)	0.5 (0.2-1.2)	0.5 (0.2-1.2)
95th percentile of interval [*], year	3.8	3.9	3.9	4.1	3.7	3.7
Median follow-up, year	11.2	11.0	11.2	11.2	11.0	11.2
Total follow-up, year	432,952	403,674	426,292	418,632	412,275	432,110

ECOPD, exacerbation of chronic obstructive pulmonary disease (COPD); IQR, interquartile range.

[*] The interval between two consecutive ECOPD admissions was analyzed only for participants with ≥2 ECOPD hospitalizations.

Table S4. Sensitivity analyses of the associations between ECOPD hospitalization and short-term CVD risk

	Sensitivity analysis 1		Sensitivity analysis 2		Sensitivity analysis 3	
	Cases	HRs (95%CIs)	Cases	HRs (95%CIs)	Cases	HRs (95%CIs)
**Acute myocardial infarction**						
Baseline	957	Reference	781	Reference	791	Reference
1-7 days	14	7.45 (4.26-13.05) *	17	7.26 (4.36-12.08) *	21	8.64 (5.42-13.76) *
8-30 days	20	3.03 (1.88-4.88) *	47	5.72 (4.09-8.00) *	46	5.34 (3.80-7.50) *
31-90 days	31	2.13 (1.43-3.16) *	52	2.73 (1.98-3.77) *	50	2.64 (1.90-3.66) *
91-365 days	81	1.77 (1.35-2.32) *	132	2.23 (1.76-2.83) *	131	2.23 (1.76-2.84) *
2nd year	42	1.21 (0.86-1.71)	86	1.95 (1.49-2.55) *	84	1.91 (1.46-2.50) *
3th year	24	1.11 (0.72-1.71)	46	1.63 (1.17-2.28) *	46	1.64 (1.17-2.29) *
4th year	20	1.40 (0.88-2.23)	26	1.35 (0.89-2.06)	26	1.35 (0.88-2.05)
**Other ischaemic heart disease**						
Baseline	5,024	Reference	4,165	Reference	4,413	Reference
1-7 days	35	6.04 (4.28-8.51) *	59	6.38 (4.88-8.33) *	64	6.74 (5.21-8.73) *
8-30 days	75	3.42 (2.69-4.35) *	163	4.64 (3.91-5.51) *	150	4.19 (3.50-5.00) *
31-90 days	90	1.76 (1.41-2.20) *	189	2.27 (1.93-2.67) *	180	2.20 (1.87-2.59) *
91-365 days	280	1.63 (1.42-1.87) *	504	1.85 (1.65-2.07) *	497	1.85 (1.65-2.07) *
2nd year	204	1.51 (1.30-1.77) *	386	1.80 (1.60-2.04) *	386	1.83 (1.62-2.06) *
3th year	125	1.45 (1.20-1.75) *	211	1.53 (1.31-1.78) *	210	1.54 (1.32-1.80) *
4th year	81	1.38 (1.10-1.74) *	126	1.32 (1.09-1.59) *	126	1.33 (1.10-1.61) *
**Heart failure**						
Baseline	868	Reference	641	Reference	647	Reference
1-7 days	13	8.58 (4.82-15.27) *	15	9.12 (5.32-15.64) *	19	10.98 (6.73-17.89) *
8-30 days	37	6.46 (4.48-9.33) *	72	11.65 (8.70-15.59) *	53	8.54 (6.17-11.81) *
31-90 days	38	2.91 (2.03-4.17) *	64	4.44 (3.29-5.99) *	58	4.10 (3.00-5.60) *
91-365 days	101	2.38 (1.85-3.06) *	136	2.89 (2.28-3.66) *	135	2.93 (2.31-3.73) *
2nd year	65	1.94 (1.45-2.60) *	91	2.47 (1.89-3.22) *	92	2.53 (1.94-3.30) *
3th year	28	1.30 (0.86-1.94)	44	1.86 (1.32-2.62) *	43	1.85 (1.31-2.61) *
4th year	22	1.55 (0.99-2.44)	37	2.32 (1.61-3.36) *	37	2.35 (1.62-3.41) *
**Pulmonary heart disease**						
Baseline	1,869	Reference	1,258	Reference	1,246	Reference
1-7 days	41	13.71 (9.84-19.12) *	79	24.03 (18.69-30.90) *	86	24.66 (19.32-31.48) *
8-30 days	97	8.73 (6.93-11.00) *	213	17.14 (14.39-20.41) *	197	15.32 (12.80-18.33) *
31-90 days	109	4.46 (3.59-5.54) *	188	6.52 (5.45-7.81) *	180	6.28 (5.23-7.54) *
91-365 days	253	3.09 (2.63-3.63) *	373	4.18 (3.61-4.83) *	363	4.08 (3.52-4.73) *

	Sensitivity analysis 1		Sensitivity analysis 2		Sensitivity analysis 3	
	Cases	HRs (95%CIs)	Cases	HRs (95%CIs)	Cases	HRs (95%CIs)
2nd year	116	1.85 (1.49-2.28) *	181	2.75 (2.30-3.29) *	181	2.76 (2.31-3.31) *
3th year	58	1.45 (1.10-1.92)	88	2.20 (1.74-2.79) *	88	2.21 (1.75-2.80) *
4th year	33	1.22 (0.85-1.74)	37	1.48 (1.05-2.08)	37	1.49 (1.06-2.10)
**Ischaemic stroke**						
Baseline	4,441	Reference	3,858	Reference	4,074	Reference
1-7 days	19	3.48 (2.19-5.53) *	27	2.85 (1.93-4.20) *	31	3.09 (2.15-4.46) *
8-30 days	48	2.36 (1.75-3.18) *	91	2.52 (2.02-3.15) *	83	2.22 (1.76-2.80) *
31-90 days	74	1.54 (1.21-1.97) *	123	1.47 (1.21-1.78) *	121	1.45 (1.19-1.76) *
91-365 days	181	1.14 (0.97-1.35)	342	1.24 (1.09-1.41) *	340	1.23 (1.08-1.41) *
2nd year	132	1.04 (0.86-1.25)	266	1.21 (1.05-1.40) *	266	1.21 (1.05-1.40)
3th year	73	0.86 (0.68-1.10)	169	1.15 (0.97-1.37)	169	1.15 (0.97-1.36)
4th year	66	1.14 (0.88-1.47)	121	1.20 (0.99-1.46)	122	1.20 (0.99-1.46)
**Intracerebral hemorrhage**						
Baseline	1,445	Reference	1,279	Reference	1,316	Reference
1-7 days	5	2.06 (0.84-5.05)	10	2.53 (1.34-4.80) *	10	2.47 (1.30-4.67) *
8-30 days	12	1.26 (0.70-2.27)	22	1.42 (0.91-2.21)	24	1.55 (1.01-2.37)
31-90 days	27	1.29 (0.86-1.94)	47	1.31 (0.95-1.81)	45	1.31 (0.95-1.82)
91-365 days	61	0.93 (0.70-1.24)	112	1.02 (0.81-1.29)	113	1.07 (0.85-1.35)
2nd year	51	1.05 (0.77-1.42)	88	1.10 (0.86-1.41)	87	1.12 (0.87-1.44)
3th year	30	1.03 (0.70-1.51)	54	1.07 (0.79-1.44)	54	1.10 (0.81-1.48)
4th year	18	0.94 (0.58-1.52)	36	1.09 (0.76-1.55)	36	1.12 (0.79-1.60)

CI, confidence interval; COPD, chronic obstructive pulmonary disease; CVD, cardiovascular disease; ECOPD, exacerbation of COPD; HR, hazard ratio; LLN, lower limit of normal.

The HRs (95% CIs) with asterisks were statistically significant after Bonferroni correction (P<0.05/6).

Three sensitivity analyses were performed: 1) using ICD-10 code J44.0-J44.1 to define ECOPD; 2) using 28 days to define the same ECOPD hospitalization course; and 3) using the LLN criterion to define screen-detected COPD patients. The multivariable models were adjusted for the same covariates as in Table 2.

Table S5. Sensitivity analyses of the associations between the first ECOPD hospitalization and long-term CVD risk

	Sensitivity analysis 1		Sensitivity analysis 2		Sensitivity analysis 3	
	Cases	HRs (95%CIs)	Cases	HRs (95%CIs)	Cases	HRs (95%CIs)
**Acute myocardial infarction**						
Baseline	924	Reference	728	Reference	738	Reference
1st year	69	1.92 (1.44-2.55) *	123	3.01 (2.36-3.85) *	123	3.02 (2.35-3.87) *
2nd year	43	1.46 (1.04-2.05)	82	2.35 (1.78-3.11) *	82	2.35 (1.78-3.12) *
3rd year	36	1.46 (1.01-2.12)	64	2.14 (1.57-2.91) *	64	2.14 (1.57-2.91) *
4th year	33	1.62 (1.10-2.38)	42	1.65 (1.15-2.36) *	41	1.61 (1.12-2.31)
5th year	30	1.87 (1.25-2.80) *	50	2.47 (1.75-3.47) *	49	2.41 (1.71-3.41) *
6th year	23	1.93 (1.23-3.03) *	37	2.50 (1.71-3.67) *	37	2.49 (1.70-3.66) *
≥7 years	31	1.34 (0.90-2.00)	61	2.00 (1.45-2.77) *	61	1.99 (1.44-2.75) *
**Other ischaemic heart disease**						
Baseline	4,893	Reference	3,898	Reference	4,146	Reference
1st year	314	2.08 (1.83-2.37) *	502	2.47 (2.21-2.77) *	494	2.47 (2.20-2.77) *
2nd year	194	1.60 (1.36-1.87) *	383	2.25 (1.98-2.55) *	383	2.28 (2.01-2.59) *
3rd year	159	1.64 (1.38-1.95) *	287	2.06 (1.79-2.37) *	282	2.06 (1.79-2.37) *
4th year	116	1.50 (1.23-1.83) *	225	1.98 (1.70-2.31) *	222	1.99 (1.70-2.32) *
5th year	87	1.51 (1.21-1.90) *	177	2.01 (1.69-2.38) *	175	2.02 (1.70-2.40) *
6th year	71	1.76 (1.38-2.26) *	125	1.99 (1.63-2.42) *	123	2.00 (1.64-2.43) *
≥7 years	80	1.19 (0.94-1.51)	206	1.82 (1.55-2.15) *	201	1.82 (1.54-2.15) *
**Heart failure**						
Baseline	835	Reference	590	Reference	596	Reference
1st year	112	3.27 (2.57-4.17) *	136	4.29 (3.38-5.44) *	131	4.23 (3.31-5.40) *
2nd year	73	2.60 (1.97-3.45) *	114	4.23 (3.27-5.46) *	111	4.22 (3.25-5.48) *
3rd year	38	1.56 (1.09-2.24)	61	2.64 (1.93-3.61) *	58	2.57 (1.87-3.55) *
4th year	40	2.08 (1.46-2.98) *	68	3.56 (2.61-4.85) *	65	3.49 (2.55-4.79) *
5th year	28	1.91 (1.26-2.89) *	52	3.52 (2.50-4.95) *	48	3.32 (2.33-4.72) *
6th year	18	1.69 (1.02-2.78)	25	2.33 (1.49-3.64) *	23	2.20 (1.38-3.49) *
≥7 years	28	1.65 (1.09-2.50)	54	2.81 (2.00-3.95) *	52	2.77 (1.96-3.92) *
**Pulmonary heart disease**						
Baseline	1,815	Reference	1,184	Reference	1,172	Reference
1st year	291	4.15 (3.56-4.84) *	397	6.15 (5.34-7.08) *	390	6.04 (5.25-6.96) *
2nd year	132	2.43 (1.99-2.98) *	218	4.30 (3.62-5.10) *	217	4.28 (3.60-5.08) *
3rd year	99	2.28 (1.81-2.86) *	174	4.28 (3.54-5.16) *	169	4.18 (3.45-5.05) *
4th year	78	2.26 (1.76-2.91) *	135	4.32 (3.50-5.32) *	128	4.11 (3.31-5.09) *
5th year	72	3.06 (2.35-3.98) *	117	5.12 (4.09-6.41) *	114	4.99 (3.97-6.26) *

	Sensitivity analysis 1		Sensitivity analysis 2		Sensitivity analysis 3	
	Cases	HRs (95%CIs)	Cases	HRs (95%CIs)	Cases	HRs (95%CIs)
6th year	40	2.48 (1.77-3.48) *	63	3.89 (2.93-5.18) *	63	3.91 (2.94-5.21) *
≥7 years	49	2.02 (1.47-2.77) *	129	5.04 (4.02-6.32) *	125	4.93 (3.92-6.20) *
**Ischaemic stroke**						
Baseline	4,322	Reference	3,618	Reference	3,835	Reference
1st year	204	1.52 (1.30-1.78) *	341	1.68 (1.47-1.92) *	338	1.66 (1.45-1.90) *
2nd year	134	1.20 (1.00-1.45)	250	1.43 (1.23-1.66) *	249	1.41 (1.21-1.64) *
3rd year	89	0.96 (0.76-1.20)	202	1.36 (1.15-1.60) *	202	1.34 (1.14-1.58) *
4th year	88	1.15 (0.92-1.45)	177	1.45 (1.22-1.73) *	175	1.42 (1.19-1.70) *
5th year	78	1.32 (1.03-1.67)	153	1.59 (1.32-1.92) *	153	1.58 (1.31-1.90) *
6th year	46	1.09 (0.80-1.47)	102	1.47 (1.18-1.83) *	101	1.44 (1.16-1.79) *
≥7 years	73	0.96 (0.75-1.23)	154	1.18 (0.98-1.42)	153	1.16 (0.96-1.40)
**Intracerebral hemorrhage**						
Baseline	1,419	Reference	1,228	Reference	1,265	Reference
1st year	62	1.11 (0.84-1.48)	97	1.12 (0.88-1.43)	97	1.16 (0.91-1.48)
2nd year	49	1.04 (0.76-1.43)	80	1.07 (0.82-1.39)	80	1.10 (0.84-1.43)
3rd year	36	0.96 (0.67-1.37)	63	0.99 (0.74-1.33)	63	1.02 (0.76-1.37)
4th year	29	0.94 (0.63-1.39)	57	1.08 (0.79-1.46)	57	1.11 (0.82-1.52)
5th year	22	0.91 (0.58-1.42)	44	1.04 (0.74-1.46)	44	1.08 (0.77-1.52)
6th year	16	0.91 (0.54-1.53)	27	0.86 (0.57-1.30)	27	0.90 (0.59-1.36)
≥7 years	16	0.55 (0.33-0.93)	52	0.95 (0.69-1.32)	52	0.99 (0.71-1.36)

CI, confidence interval; COPD, chronic obstructive pulmonary disease; CVD, cardiovascular disease; ECOPD, exacerbation of COPD; HR, hazard ratio; LLN, lower limit of normal.

The HRs (95% CIs) with asterisks were statistically significant after Bonferroni correction ($P<0.05/6$).

Three sensitivity analyses were performed: 1) using ICD-10 code J44.0-J44.1 to define ECOPD; 2) using 28 days to define the same ECOPD hospitalization course; and 3) using the LLN criterion to define screen-detected COPD patients. The multivariable models were adjusted for the same covariates as in Table 2.

Table S6. Associations between ECOPD hospitalization and short-term CVD risk stratified by age

	<60 years		≥60 years		P_{int}
	Cases	HRs (95%CIs)	Cases	HRs (95%CIs)	
**Acute myocardial infarction**					0.026
Baseline	173	Reference	608	Reference	
1-30 days	12	6.86 (3.73-12.63) *	57	5.14 (3.88-6.82) *	
31-365 days	37	2.94 (2.00-4.30) *	142	1.89 (1.55-2.29) *	
2nd years	18	2.35 (1.42-3.88) *	66	1.55 (1.19-2.02) *	
3-4 years	22	2.50 (1.58-3.95) *	50	1.09 (0.81-1.46)	
**Other ischaemic heart disease**					0.003 *
Baseline	1,387	Reference	2,778	Reference	
1-30 days	72	6.67 (5.22-8.51) *	149	4.21 (3.56-4.99) *	
31-365 days	193	2.30 (1.96-2.70) *	477	1.82 (1.64-2.01) *	
2nd years	110	2.02 (1.66-2.47) *	276	1.77 (1.56-2.01) *	
3-4 years	95	1.51 (1.22-1.87) *	241	1.43 (1.25-1.64) *	
**Heart failure**					0.005 *
Baseline	155	Reference	486	Reference	
1-30 days	23	16.00 (10.00-25.60) *	49	6.86 (5.06-9.31) *	
31-365 days	42	3.86 (2.67-5.57) *	151	2.94 (2.42-3.58) *	
2nd years	22	3.36 (2.11-5.35) *	70	2.29 (1.77-2.97) *	
3-4 years	24	3.24 (2.07-5.07) *	56	1.69 (1.27-2.26) *	
**Pulmonary heart disease**					<0.001 *
Baseline	285	Reference	973	Reference	
1-30 days	74	29.45 (22.10-39.23) *	218	18.01 (15.37-21.11) *	
31-365 days	155	8.51 (6.81-10.64) *	379	4.56 (4.00-5.19) *	
2nd years	46	4.41 (3.17-6.12) *	135	2.86 (2.37-3.46) *	
3-4 years	37	3.52 (2.45-5.05) *	88	1.92 (1.53-2.40) *	
**Ischaemic stroke**					0.341
Baseline	1,298	Reference	2,559	Reference	
1-30 days	30	2.75 (1.90-3.97) *	86	2.34 (1.88-2.92) *	
31-365 days	112	1.31 (1.07-1.60)	347	1.31 (1.16-1.47) *	
2nd years	83	1.48 (1.18-1.86) *	183	1.15 (0.99-1.34)	
3-4 years	79	1.18 (0.94-1.49)	212	1.22 (1.06-1.41)	
**Intracerebral haemorrhage**					0.212
Baseline	354	Reference	925	Reference	
1-30 days	8	2.29 (1.12-4.68)	27	1.50 (1.02-2.22)	
31-365 days	36	1.38 (0.96-1.98)	121	1.02 (0.83-1.24)	
2nd years	26	1.63 (1.08-2.46)	61	0.93 (0.71-1.21)	
3-4 years	18	1.02 (0.63-1.65)	72	1.08 (0.84-1.38)	

CI, confidence interval; CVD, cardiovascular disease; ECOPD, exacerbation of chronic obstructive pulmonary disease (COPD); HR, hazard ratio.

The HRs (95% CIs) with asterisks were statistically significant after Bonferroni correction (P<0.05/12), so were the P_{int} (<0.05/6).

The multivariable models were adjusted for the same covariates as in Table 2.

Table S7. Associations between first ECOPD hospitalization and long-term CVD risk stratified by age

	<60 years		≥60 years		P_{int}
	Cases	HRs (95%CIs)	Cases	HRs (95%CIs)	
**Acute myocardial infarction**					0.064
Baseline	163	Reference	565	Reference	
1-2 years	47	3.23 (2.29-4.56) *	158	2.02 (1.67-2.43) *	
3-4 years	25	2.37 (1.52-3.69) *	80	1.36 (1.06-1.74)	
5-6 years	18	2.55 (1.52-4.25) *	68	1.81 (1.39-2.37) *	
≥7 years	9	1.54 (0.77-3.11)	52	1.64 (1.21-2.22) *	
**Other ischaemic heart disease**					0.003 *
Baseline	1,298	Reference	2,600	Reference	
1-2 years	272	2.66 (2.32-3.05) *	605	2.01 (1.83-2.21) *	
3-4 years	132	1.86 (1.54-2.24) *	372	1.81 (1.62-2.03) *	
5-6 years	85	1.94 (1.54-2.43) *	213	1.75 (1.51-2.02) *	
≥7 years	70	1.96 (1.52-2.51) *	131	1.48 (1.23-1.78) *	
**Heart failure**					0.021
Baseline	140	Reference	450	Reference	
1-2 years	62	5.36 (3.89-7.39) *	180	3.25 (2.71-3.91) *	
3-4 years	33	3.93 (2.62-5.91) *	90	2.23 (1.76-2.84) *	
5-6 years	17	3.35 (1.97-5.71) *	54	2.19 (1.62-2.96) *	
≥7 years	14	3.43 (1.89-6.19) *	38	2.20 (1.54-3.14) *	
**Pulmonary heart disease**					<0.001 *
Baseline	265	Reference	919	Reference	
1-2 years	173	8.83 (7.13-10.93) *	434	4.77 (4.22-5.40) *	
3-4 years	73	5.89 (4.42-7.85) *	224	4.02 (3.42-4.72) *	
5-6 years	46	6.24 (4.40-8.86) *	131	4.46 (3.64-5.46) *	
≥7 years	40	8.27 (5.64-12.13) *	85	4.77 (3.72-6.12) *	
**Ischaemic stroke**					0.721
Baseline	1,221	Reference	2,397	Reference	
1-2 years	163	1.55 (1.31-1.84) *	424	1.42 (1.27-1.58) *	
3-4 years	103	1.36 (1.10-1.67) *	274	1.29 (1.13-1.47) *	
5-6 years	72	1.53 (1.19-1.95) *	182	1.42 (1.22-1.67) *	
≥7 years	43	1.07 (0.78-1.47)	110	1.14 (0.94-1.40)	
**Intracerebral haemorrhage**					0.367
Baseline	338	Reference	890	Reference	
1-2 years	42	1.36 (0.97-1.89)	135	1.05 (0.87-1.26)	
3-4 years	30	1.39 (0.94-2.05)	90	0.97 (0.77-1.22)	
5-6 years	19	1.41 (0.88-2.28)	52	0.90 (0.67-1.20)	
≥7 years	13	1.25 (0.70-2.22)	39	0.91 (0.65-1.27)	

CI, confidence interval; CVD, cardiovascular disease; ECOPD, exacerbation of chronic obstructive pulmonary disease (COPD); HR, hazard ratio.

The HRs (95% CIs) with asterisks were statistically significant after Bonferroni correction ($P<0.05/12$), so were the P_{int} (<0.05/6).

The multivariable models were adjusted for the same covariates as in Table 2.

Table S8. Associations between ECOPD hospitalization and short-term CVD risk stratified by sex

	Women		Men		P_{int}
	Cases	HRs (95%CIs)	Cases	HRs (95%CIs)	
**Acute myocardial infarction**					0.200
Baseline	310	Reference	471	Reference	
1-30 days	29	7.10 (4.50-11.22) *	40	5.48 (3.74-8.05) *	
31-365 days	69	2.25 (1.58-3.20) *	110	2.31 (1.74-3.06) *	
2nd years	37	1.92 (1.27-2.90) *	47	1.84 (1.29-2.63) *	
3-4 years	38	1.77 (1.18-2.66)	34	1.24 (0.83-1.84)	
**Other ischaemic heart disease**					0.057
Baseline	2,186	Reference	1,979	Reference	
1-30 days	79	3.86 (3.02-4.94) *	142	5.26 (4.32-6.41) *	
31-365 days	292	1.79 (1.54-2.09) *	378	2.00 (1.73-2.31) *	
2nd years	202	1.85 (1.56-2.20) *	184	1.76 (1.47-2.10) *	
3-4 years	190	1.53 (1.29-1.82) *	146	1.32 (1.09-1.60)	
**Heart failure**					0.252
Baseline	307	Reference	334	Reference	
1-30 days	29	9.83 (6.27-15.41) *	43	7.69 (5.22-11.32) *	
31-365 days	90	3.83 (2.78-5.28) *	103	2.68 (1.99-3.63) *	
2nd years	51	3.36 (2.33-4.84) *	41	1.87 (1.28-2.75) *	
3-4 years	44	2.37 (1.61-3.48) *	36	1.65 (1.11-2.47)	
**Pulmonary heart disease**					0.514
Baseline	489	Reference	769	Reference	
1-30 days	105	20.19 (15.47-26.35) *	187	15.69 (12.81-19.23) *	
31-365 days	180	4.57 (3.65-5.71) *	354	4.58 (3.86-5.43) *	
2nd years	71	2.93 (2.19-3.92) *	110	2.69 (2.13-3.39) *	
3-4 years	54	2.04 (1.48-2.82) *	71	1.83 (1.39-2.40) *	
**Ischaemic stroke**					0.494
Baseline	1,779	Reference	2,078	Reference	
1-30 days	53	2.69 (2.00-3.63) *	63	2.21 (1.68-2.91) *	
31-365 days	205	1.32 (1.11-1.58) *	254	1.25 (1.07-1.48)	
2nd years	121	1.19 (0.96-1.48)	145	1.26 (1.04-1.53)	
3-4 years	143	1.13 (0.93-1.38)	148	1.21 (1.00-1.47)	
**Intracerebral haemorrhage**					0.243
Baseline	501	Reference	778	Reference	
1-30 days	14	1.98 (1.11-3.53)	21	1.45 (0.91-2.32)	
31-365 days	65	1.31 (0.95-1.82)	92	0.98 (0.75-1.29)	
2nd years	43	1.45 (1.01-2.10)	44	0.89 (0.63-1.26)	
3-4 years	38	1.13 (0.77-1.65)	52	1.03 (0.75-1.43)	

CI, confidence interval; CVD, cardiovascular disease; ECOPD, exacerbation of chronic obstructive pulmonary disease (COPD); HR, hazard ratio.

The HRs (95% CIs) with asterisks were statistically significant after Bonferroni correction (P<0.05/12), so were the P_{int} (<0.05/6).

The multivariable models were adjusted for all other covariates except sex, as shown in Table 2.

Table S9. Associations between first ECOPD hospitalization and long-term CVD risk stratified by sex

	Women		Men		P_{int}
	Cases	HRs (95%CIs)	Cases	HRs (95%CIs)	
**Acute myocardial infarction**					0.365
Baseline	283	Reference	445	Reference	
1-2 years	87	2.82 (2.00-3.98) *	118	2.42 (1.84-3.20) *	
3-4 years	49	2.03 (1.35-3.04) *	56	1.63 (1.15-2.32)	
5-6 years	37	2.40 (1.54-3.76) *	49	2.29 (1.56-3.35) *	
≥7 years	27	1.91 (1.16-3.13)	34	1.93 (1.25-2.98) *	
**Other ischaemic heart disease**					0.253
Baseline	2,038	Reference	1,860	Reference	
1-2 years	392	2.13 (1.85-2.46) *	485	2.47 (2.16-2.82) *	
3-4 years	261	2.03 (1.72-2.39) *	243	1.90 (1.60-2.25) *	
5-6 years	155	1.98 (1.62-2.41) *	143	1.89 (1.54-2.32) *	
≥7 years	103	1.65 (1.31-2.08) *	98	1.87 (1.47-2.37) *	
**Heart failure**					0.274
Baseline	282	Reference	308	Reference	
1-2 years	120	4.70 (3.47-6.35) *	122	3.45 (2.58-4.60) *	
3-4 years	62	3.15 (2.18-4.54) *	61	2.49 (1.74-3.58) *	
5-6 years	30	2.38 (1.50-3.77) *	41	2.93 (1.92-4.45) *	
≥7 years	27	2.78 (1.72-4.51) *	25	2.51 (1.53-4.14) *	
**Pulmonary heart disease**					0.850
Baseline	458	Reference	726	Reference	
1-2 years	216	5.37 (4.36-6.63) *	391	5.01 (4.26-5.90) *	
3-4 years	110	4.13 (3.17-5.38) *	187	3.93 (3.19-4.83) *	
5-6 years	64	4.32 (3.13-5.98) *	113	4.37 (3.39-5.63) *	
≥7 years	51	4.52 (3.14-6.50) *	74	4.97 (3.69-6.70) *	
**Ischaemic stroke**					0.193
Baseline	1,641	Reference	1,977	Reference	
1-2 years	278	1.70 (1.44-2.02) *	309	1.41 (1.21-1.65) *	
3-4 years	175	1.44 (1.18-1.75) *	202	1.34 (1.12-1.61) *	
5-6 years	127	1.63 (1.30-2.03) *	127	1.41 (1.13-1.75) *	
≥7 years	80	1.15 (0.89-1.50)	73	1.17 (0.89-1.52)	
**Intracerebral haemorrhage**					0.305
Baseline	480	Reference	748	Reference	
1-2 years	77	1.31 (0.96-1.80)	100	0.99 (0.76-1.29)	
3-4 years	45	1.00 (0.69-1.47)	75	1.05 (0.78-1.42)	
5-6 years	35	1.21 (0.79-1.86)	36	0.81 (0.55-1.21)	
≥7 years	24	0.97 (0.59-1.57)	28	0.91 (0.59-1.41)	

CI, confidence interval; CVD, cardiovascular disease; ECOPD, exacerbation of chronic obstructive pulmonary disease (COPD); HR, hazard ratio.

The HRs (95% CIs) with asterisks were statistically significant after Bonferroni correction (P<0.05/12), so were the P_{int} (<0.05/6).

The multivariable models were adjusted for all other covariates except sex, as shown in Table 2.

Table S10. Associations between ECOPD hospitalization and short-term CVD risk stratified by study region

	Urban		Rural		P_{int}
	Cases	HRs (95%CIs)	Cases	HRs (95%CIs)	
**Acute myocardial infarction**					0.064
Baseline	201	Reference	580	Reference	
1-30 days	8	4.46 (2.06-9.67) *	61	6.48 (4.71-8.93) *	
31-365 days	30	2.28 (1.41-3.68) *	149	2.29 (1.79-2.93) *	
2nd years	24	2.79 (1.67-4.66) *	60	1.68 (1.23-2.30) *	
3-4 years	10	0.90 (0.45-1.79)	62	1.67 (1.23-2.28) *	
**Other ischaemic heart disease**					<0.001 *
Baseline	1,552	Reference	2,613	Reference	
1-30 days	26	2.27 (1.51-3.41) *	195	5.68 (4.80-6.73) *	
31-365 days	154	1.66 (1.37-2.02) *	516	2.07 (1.83-2.34) *	
2nd years	96	1.49 (1.18-1.88) *	290	2.00 (1.73-2.31) *	
3-4 years	98	1.27 (1.01-1.60)	238	1.56 (1.33-1.82) *	
**Heart failure**					0.552
Baseline	196	Reference	445	Reference	
1-30 days	12	5.53 (2.87-10.63) *	60	10.18 (7.32-14.16) *	
31-365 days	53	2.93 (1.95-4.39) *	140	3.35 (2.58-4.33) *	
2nd years	21	1.69 (1.00-2.88)	71	2.92 (2.15-3.96) *	
3-4 years	23	1.61 (0.97-2.69)	57	2.18 (1.57-3.03) *	
**Pulmonary heart disease**					0.185
Baseline	153	Reference	1,105	Reference	
1-30 days	35	18.48 (11.57-29.52) *	257	17.11 (14.44-20.29) *	
31-365 days	69	5.21 (3.55-7.66) *	465	4.52 (3.92-5.22) *	
2nd years	34	4.47 (2.83-7.08) *	147	2.56 (2.10-3.11) *	
3-4 years	16	2.01 (1.12-3.60)	109	1.92 (1.54-2.39) *	
**Ischaemic stroke**					0.376
Baseline	1,667	Reference	2,190	Reference	
1-30 days	30	2.21 (1.51-3.23) *	86	2.36 (1.86-3.00) *	
31-365 days	166	1.56 (1.29-1.89) *	293	1.13 (0.97-1.32)	
2nd years	100	1.41 (1.12-1.76) *	166	1.10 (0.91-1.32)	
3-4 years	111	1.29 (1.04-1.61)	180	1.10 (0.92-1.31)	
**Intracerebral haemorrhage**					0.244
Baseline	252	Reference	1,027	Reference	
1-30 days	2	0.86 (0.20-3.58)	33	1.78 (1.22-2.60) *	
31-365 days	25	1.56 (0.95-2.58)	132	1.04 (0.83-1.31)	
2nd years	10	1.00 (0.50-1.99)	77	1.11 (0.85-1.45)	
3-4 years	11	0.85 (0.44-1.67)	79	1.12 (0.86-1.46)	

CI, confidence interval; CVD, cardiovascular disease; ECOPD, exacerbation of chronic obstructive pulmonary disease (COPD); HR, hazard ratio.

The HRs (95% CIs) with asterisks were statistically significant after Bonferroni correction (P<0.05/12), so were the P_{int} (<0.05/6).

The multivariable models were adjusted for the same covariates as in Table 2.

Table S11. Associations between first ECOPD hospitalization and long-term CVD risk stratified by study region

	Urban		Rural		P_{int}
	Cases	HRs (95%CIs)	Cases	HRs (95%CIs)	
**Acute myocardial infarction**					0.102
Baseline	186	Reference	542	Reference	
1-2 years	44	2.48 (1.59-3.87) *	161	2.65 (2.07-3.38) *	
3-4 years	14	0.98 (0.51-1.86)	91	2.09 (1.56-2.79) *	
5-6 years	13	1.36 (0.69-2.65)	73	2.67 (1.93-3.68) *	
≥7 years	16	1.38 (0.71-2.67)	45	2.15 (1.48-3.13) *	
**Other ischaemic heart disease**					<0.001 *
Baseline	1,466	Reference	2,432	Reference	
1-2 years	192	1.64 (1.36-1.97) *	685	2.75 (2.45-3.09) *	
3-4 years	136	1.64 (1.33-2.03) *	368	2.22 (1.93-2.56) *	
5-6 years	81	1.60 (1.23-2.08) *	217	2.21 (1.86-2.62) *	
≥7 years	51	1.12 (0.82-1.54)	150	2.24 (1.84-2.72) *	
**Heart failure**					0.606
Baseline	178	Reference	412	Reference	
1-2 years	63	3.42 (2.30-5.10) *	179	4.27 (3.34-5.45) *	
3-4 years	26	1.80 (1.07-3.03)	97	3.31 (2.47-4.45) *	
5-6 years	21	2.29 (1.29-4.05)	50	2.89 (2.01-4.17) *	
≥7 years	17	2.29 (1.22-4.28)	35	2.88 (1.90-4.37) *	
**Pulmonary heart disease**					0.473
Baseline	146	Reference	1,038	Reference	
1-2 years	84	5.57 (3.88-7.99) *	523	5.13 (4.48-5.88) *	
3-4 years	41	3.95 (2.50-6.23) *	256	4.08 (3.43-4.85) *	
5-6 years	20	3.26 (1.82-5.84) *	157	4.64 (3.76-5.73) *	
≥7 years	16	3.49 (1.82-6.71) *	109	5.15 (4.04-6.58) *	
**Ischaemic stroke**					
Baseline	1,575	Reference	2,043	Reference	0.312
1-2 years	228	1.77 (1.49-2.10) *	359	1.40 (1.20-1.63) *	
3-4 years	135	1.50 (1.22-1.85) *	242	1.31 (1.11-1.56) *	
5-6 years	85	1.55 (1.20-2.00) *	169	1.50 (1.23-1.82) *	
≥7 years	51	1.13 (0.83-1.55)	102	1.19 (0.94-1.50)	
**Intracerebral haemorrhage**					0.538
Baseline	240	Reference	988	Reference	
1-2 years	30	1.37 (0.84-2.22)	147	1.07 (0.85-1.33)	
3-4 years	16	0.99 (0.54-1.82)	104	1.05 (0.81-1.36)	
5-6 years	8	0.74 (0.33-1.66)	63	1.02 (0.75-1.38)	
≥7 years	6	0.75 (0.30-1.85)	46	1.00 (0.71-1.42)	

CI, confidence interval; CVD, cardiovascular disease; ECOPD, exacerbation of chronic obstructive pulmonary disease (COPD); HR, hazard ratio.

The HRs (95% CIs) with asterisks were statistically significant after Bonferroni correction ($P<0.05/12$), so were the P_{int} (<0.05/6).

The multivariable models were adjusted for the same covariates as in Table 2.

Table S12. Associations between ECOPD hospitalization and short-term CVD risk stratified by smoking status

	Never-smoker		Ever-smoker		P_{int}
	Cases	HRs (95%CIs)	Cases	HRs (95%CIs)	
**Acute myocardial infarction**					0.007 *
Baseline	372	Reference	409	Reference	
1-30 days	31	8.11 (5.26-12.51) *	38	5.15 (3.46-7.67) *	
31-365 days	70	2.35 (1.68-3.28) *	109	2.24 (1.67-3.00) *	
2nd years	41	2.19 (1.49-3.23) *	43	1.66 (1.14-2.40)	
3-4 years	44	2.01 (1.37-2.93) *	28	1.05 (0.68-1.61)	
**Other ischaemic heart disease**					0.078
Baseline	2,379	Reference	1,786	Reference	
1-30 days	76	3.83 (2.99-4.91) *	145	5.30 (4.35-6.47) *	
31-365 days	296	1.83 (1.58-2.13) *	374	1.97 (1.70-2.28) *	
2nd years	200	1.83 (1.55-2.17) *	186	1.80 (1.50-2.14) *	
3-4 years	190	1.53 (1.28-1.81) *	146	1.34 (1.11-1.63) *	
**Heart failure**					0.004 *
Baseline	324	Reference	317	Reference	
1-30 days	28	10.01 (6.37-15.72) *	44	7.72 (5.25-11.36) *	
31-365 days	95	4.22 (3.09-5.76) *	98	2.43 (1.79-3.30) *	
2nd years	47	3.25 (2.24-4.71) *	45	1.98 (1.36-2.89) *	
3-4 years	49	2.72 (1.88-3.94) *	31	1.33 (0.87-2.04)	
**Pulmonary heart disease**					0.838
Baseline	515	Reference	743	Reference	
1-30 days	94	18.39 (13.98-24.18) *	198	16.47 (13.48-20.14) *	
31-365 days	179	4.76 (3.82-5.94) *	355	4.43 (3.73-5.25) *	
2nd years	62	2.69 (1.98-3.64) *	119	2.72 (2.17-3.41) *	
3-4 years	53	2.16 (1.56-2.98) *	72	1.74 (1.33-2.28) *	
**Ischaemic stroke**					0.046
Baseline	2,102	Reference	1,755	Reference	
1-30 days	46	2.47 (1.80-3.38) *	70	2.38 (1.83-3.10) *	
31-365 days	225	1.50 (1.26-1.77) *	234	1.13 (0.95-1.34)	
2nd years	120	1.19 (0.97-1.47)	146	1.24 (1.02-1.51)	
3-4 years	139	1.10 (0.90-1.34)	152	1.25 (1.03-1.52)	
**Intracerebral haemorrhage**					0.237
Baseline	592	Reference	687	Reference	
1-30 days	15	2.10 (1.20-3.65)	20	1.37 (0.84-2.22)	
31-365 days	63	1.23 (0.89-1.69)	94	1.01 (0.77-1.33)	
2nd years	41	1.32 (0.91-1.90)	46	0.95 (0.67-1.33)	
3-4 years	46	1.29 (0.91-1.84)	44	0.89 (0.62-1.25)	

CI, confidence interval; CVD, cardiovascular disease; ECOPD, exacerbation of chronic obstructive pulmonary disease (COPD); HR, hazard ratio.

The HRs (95% CIs) with asterisks were statistically significant after Bonferroni correction (P<0.05/12), so were the P_{int} (<0.05/6).

The multivariable models were adjusted for all other covariates except tobacco smoking, as shown in Table 2.

Table S13. Associations between first ECOPD hospitalization and long-term CVD risk stratified by smoking status

	Never-smoker		Ever-smoker		P_{int}
	Cases	HRs (95%CIs)	Cases	HRs (95%CIs)	
**Acute myocardial infarction**					0.021
Baseline	346	Reference	382	Reference	
1-2 years	93	2.88 (2.09-3.96) *	112	2.44 (1.82-3.26) *	
3-4 years	57	2.31 (1.59-3.35) *	48	1.46 (1.00-2.12)	
5-6 years	32	2.01 (1.29-3.15) *	54	2.63 (1.79-3.85) *	
≥7 years	30	2.18 (1.37-3.47) *	31	1.83 (1.16-2.90)	
**Other ischaemic heart disease**					0.057
Baseline	2,226	Reference	1,672	Reference	
1-2 years	391	2.11 (1.84-2.43) *	486	2.50 (2.18-2.87) *	
3-4 years	264	2.06 (1.75-2.42) *	240	1.88 (1.58-2.23) *	
5-6 years	163	2.07 (1.70-2.51) *	135	1.81 (1.46-2.23) *	
≥7 years	97	1.59 (1.26-2.01) *	104	1.92 (1.52-2.43) *	
**Heart failure**					0.005 *
Baseline	295	Reference	295	Reference	
1-2 years	121	5.35 (3.98-7.20) *	121	3.06 (2.28-4.10) *	
3-4 years	69	3.97 (2.79-5.65) *	54	1.94 (1.33-2.83) *	
5-6 years	33	3.07 (1.97-4.78) *	38	2.39 (1.55-3.67) *	
≥7 years	25	3.05 (1.86-4.98) *	27	2.30 (1.41-3.75) *	
**Pulmonary heart disease**					0.421
Baseline	480	Reference	704	Reference	
1-2 years	215	5.65 (4.59-6.95) *	392	4.84 (4.11-5.70) *	
3-4 years	102	4.15 (3.18-5.43) *	195	3.88 (3.16-4.78) *	
5-6 years	60	4.30 (3.09-5.98) *	117	4.38 (3.41-5.64) *	
≥7 years	46	4.63 (3.18-6.74) *	79	4.92 (3.67-6.60) *	
**Ischaemic stroke**					0.236
Baseline	1,964	Reference	1,654	Reference	
1-2 years	294	1.72 (1.47-2.02) *	293	1.38 (1.17-1.62) *	
3-4 years	173	1.38 (1.14-1.68) *	204	1.40 (1.16-1.68) *	
5-6 years	124	1.57 (1.26-1.95) *	130	1.50 (1.20-1.87) *	
≥7 years	77	1.14 (0.88-1.48)	76	1.21 (0.92-1.58)	
**Intracerebral haemorrhage**					0.456
Baseline	569	Reference	659	Reference	
1-2 years	79	1.26 (0.93-1.71)	98	1.00 (0.76-1.30)	
3-4 years	55	1.19 (0.84-1.69)	65	0.91 (0.66-1.25)	
5-6 years	32	1.08 (0.71-1.66)	39	0.88 (0.60-1.30)	
≥7 years	22	0.99 (0.61-1.62)	30	0.91 (0.59-1.40)	

CI, confidence interval; CVD, cardiovascular disease; ECOPD, exacerbation of chronic obstructive pulmonary disease (COPD); HR, hazard ratio.

The HRs (95% CIs) with asterisks were statistically significant after Bonferroni correction (P<0.05/12), so were the P_{int} (<0.05/6).

The multivariable models were adjusted for all other covariates except tobacco smoking, as shown in Table 2.

	Cases	HRs (95%CIs)
**Acute myocardial infarction**		
Baseline	728	Reference
1st year	104	5.14 (3.92-6.75) *
2nd year	51	3.36 (2.39-4.72) *
3rd year	36	2.99 (2.03-4.42) *
4th year	19	2.04 (1.24-3.36) *
5th year	14	2.05 (1.16-3.63)
6th year	14	3.09 (1.74-5.48) *
≥7 years	18	2.00 (1.19-3.36)
**Heart failure**		
Baseline	590	Reference
1st year	106	6.26 (4.78-8.21) *
2nd year	59	4.69 (3.39-6.49) *
3rd year	29	2.96 (1.94-4.52) *
4th year	30	3.92 (2.57-5.98) *
5th year	22	4.19 (2.59-6.75) *
6th year	6	1.71 (0.74-3.94)
≥7 years	15	2.56 (1.47-4.46) *
**Ischaemic stroke**		
Baseline	3,618	Reference
1st year	297	2.50 (2.16-2.89) *
2nd year	177	1.92 (1.61-2.29) *
3rd year	120	1.67 (1.36-2.05) *
4th year	95	1.70 (1.35-2.13) *
5th year	88	2.13 (1.68-2.69) *
6th year	53	1.90 (1.42-2.54) *
≥7 years	68	1.42 (1.10-1.84) *

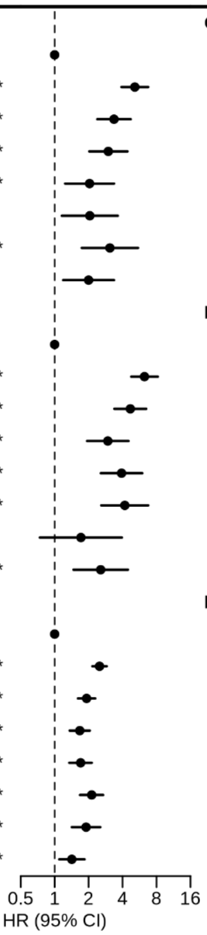

	Cases	HRs (95%CIs)
**Other ischaemic heart disease**		
Baseline	3,898	Reference
1st year	416	3.63 (3.20-4.12) *
2nd year	260	3.00 (2.59-3.48) *
3rd year	143	2.19 (1.82-2.64) *
4th year	98	1.92 (1.54-2.39) *
5th year	91	2.42 (1.93-3.04) *
6th year	56	2.21 (1.67-2.92) *
≥7 years	91	2.11 (1.68-2.65) *
**Pulmonary heart disease**		
Baseline	1,184	Reference
1st year	289	9.30 (7.90-10.96) *
2nd year	96	4.49 (3.55-5.68) *
3rd year	57	3.77 (2.82-5.05) *
4th year	28	2.70 (1.81-4.02) *
5th year	28	3.84 (2.55-5.78) *
6th year	12	2.29 (1.25-4.20) *
≥7 years	19	2.66 (1.64-4.31) *
**Intracerebral haemorrhage**		
Baseline	1,228	Reference
1st year	84	1.94 (1.49-2.53) *
2nd year	53	1.62 (1.18-2.22) *
3rd year	32	1.29 (0.88-1.90)
4th year	28	1.47 (0.97-2.22)
5th year	13	0.92 (0.52-1.63)
6th year	9	0.91 (0.46-1.80)
≥7 years	18	1.17 (0.71-1.93)

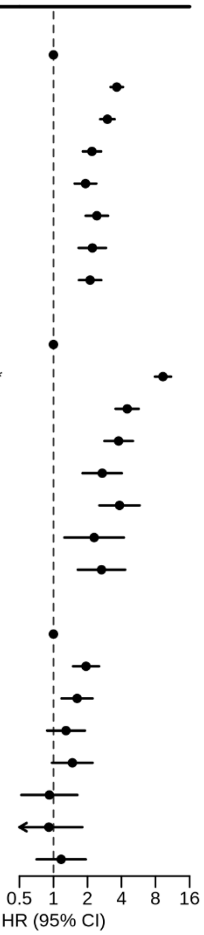

Figure S1. Associations between first ECOPD hospitalization and long-term CVD risk in patients with only one hospitalization during follow-up

CI, confidence interval; CVD, cardiovascular disease; ECOPD, exacerbation of chronic obstructive pulmonary disease (COPD); HR, hazard ratio.

The HRs (95% CIs) with asterisks were statistically significant after Bonferroni correction (*P*<0.05/6).

The multivariable models were adjusted for the same covariates as in Table 2.

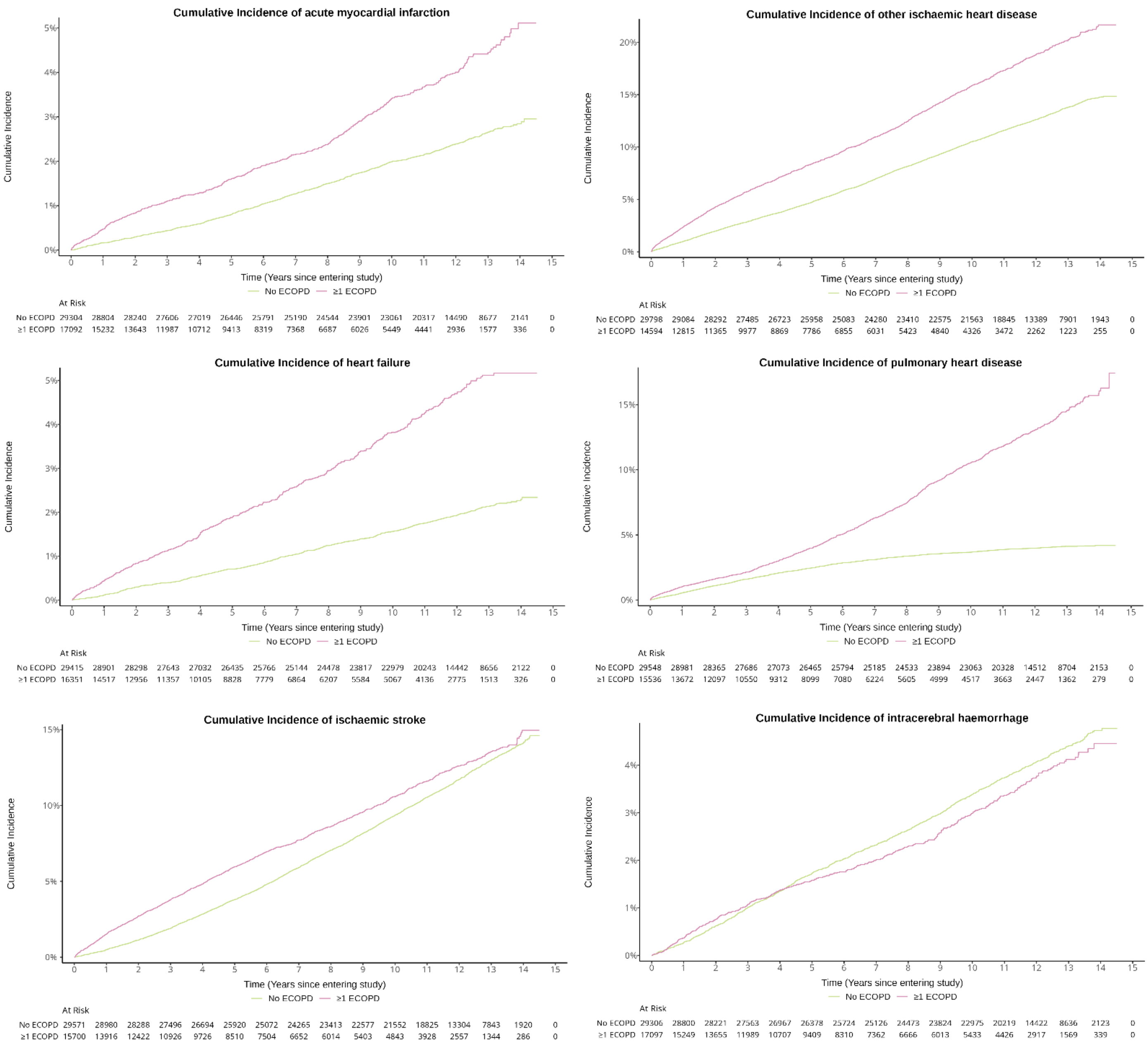

Figure S2. Cumulative incidence of CVD outcomes by the number of ECOPD

CVD, cardiovascular disease; ECOPD, exacerbation of chronic obstructive pulmonary disease (COPD).

	Cases	Competing events	SHRs (95%CIs)
**Acute myocardial infarction**			
Baseline	781	6,318	Reference
1-7 days	21	211	5.73 (3.61-9.10) *
8-30 days	46	547	3.98 (2.87-5.52) *
31-90 days	50	555	2.26 (1.65-3.10) *
91-365 days	131	1,034	2.00 (1.60-2.51) *
2nd year	84	594	1.73 (1.33-2.25) *
3th year	46	346	1.52 (1.09-2.12)
4th year	26	205	1.25 (0.83-1.89)
**Heart failure**			
Baseline	641	6,618	Reference
1-7 days	19	199	6.65 (4.08-10.82) *
8-30 days	53	503	5.86 (4.26-8.05) *
31-90 days	58	505	3.38 (2.47-4.63) *
91-365 days	135	979	2.55 (2.00-3.25) *
2nd year	92	577	2.23 (1.71-2.91) *
3th year	43	342	1.63 (1.15-2.32) *
4th year	37	193	2.11 (1.46-3.06) *
**Ischaemic stroke**			
Baseline	3,857	5,740	Reference
1-7 days	31	201	2.04 (1.40-2.96) *
8-30 days	83	524	1.67 (1.33-2.10) *
31-90 days	121	511	1.30 (1.07-1.58)
91-365 days	340	948	1.15 (1.01-1.32)
2nd year	266	531	1.16 (1.01-1.34)
3th year	169	299	1.12 (0.95-1.33)
4th year	122	165	1.18 (0.97-1.43)

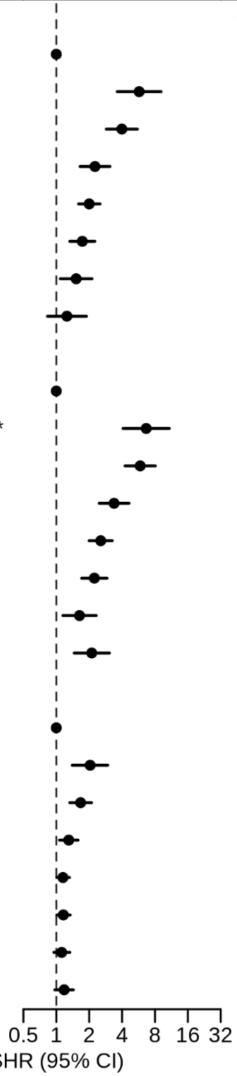

	Cases	Competing events	SHRs (95%CIs)
**Other ischaemic heart disease**			
Baseline	4,165	5,553	Reference
1-7 days	64	171	4.31 (3.30-5.63) *
8-30 days	150	394	3.19 (2.67-3.80) *
31-90 days	180	409	1.93 (1.64-2.28) *
91-365 days	497	757	1.71 (1.52-1.91) *
2nd year	386	429	1.71 (1.52-1.94) *
3th year	210	249	1.46 (1.25-1.70) *
4th year	126	137	1.27 (1.05-1.54)
**Pulmonary heart disease**			
Baseline	1,258	6,042	Reference
1-7 days	86	113	17.41 (13.44-22.56) *
8-30 days	197	274	12.28 (10.15-14.85) *
31-90 days	180	317	5.68 (4.73-6.83) *
91-365 days	363	705	3.80 (3.26-4.42) *
2nd year	181	444	2.61 (2.17-3.13) *
3th year	88	269	2.10 (1.65-2.67) *
4th year	37	169	1.43 (1.01-2.01)
**Intracerebral haemorrhage**			
Baseline	1,279	5,949	Reference
1-7 days	10	218	1.68 (0.88-3.18)
8-30 days	24	565	1.17 (0.77-1.78)
31-90 days	45	562	1.17 (0.85-1.60)
91-365 days	113	1,053	0.99 (0.79-1.24)
2nd year	87	596	1.05 (0.82-1.34)
3th year	54	332	1.04 (0.77-1.40)
4th year	36	205	1.06 (0.74-1.53)

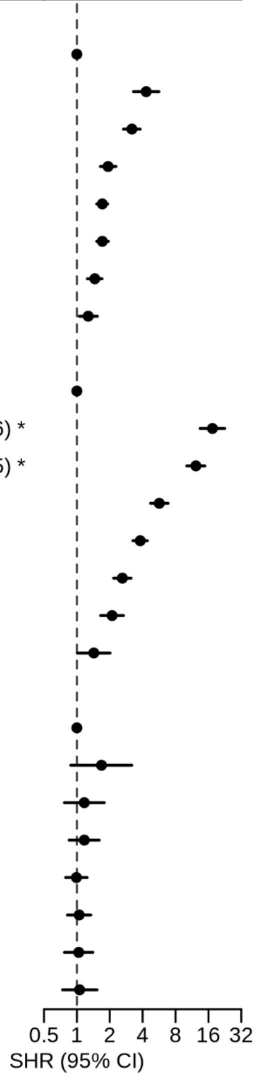

Figure S3. Subdistribution hazard ratios and 95% confidence intervals for short-term CVD risk after ECOPD hospitalization

CI, confidence interval; CVD, cardiovascular disease; ECOPD, exacerbation of chronic obstructive pulmonary disease (COPD); SHR, subdistribution hazard ratio.

The SHRs (95% CIs) with asterisks were statistically significant after Bonferroni correction (*P*<0.05/6).

Models were adjusted for the same covariates as in Table 2.

	Cases	Competing events	SHRs (95%CIs)
**Acute myocardial infarction**			
Baseline	728	5,981	Reference
1st year	123	1,119	2.39 (1.90-3.00) *
2nd year	82	663	1.98 (1.52-2.59) *
3rd year	64	547	1.87 (1.38-2.52) *
4th year	41	426	1.44 (1.02-2.05)
5th year	49	341	2.19 (1.55-3.07) *
6th year	37	258	2.25 (1.54-3.27) *
≥7 years	61	475	1.89 (1.38-2.60) *
**Heart failure**			
Baseline	590	6,276	Reference
1st year	131	1,112	3.18 (2.52-4.02) *
2nd year	111	632	3.47 (2.70-4.47) *
3rd year	58	522	2.16 (1.58-2.95) *
4th year	65	389	3.02 (2.21-4.12) *
5th year	48	320	2.86 (2.03-4.04) *
6th year	23	233	1.93 (1.22-3.04) *
≥7 years	52	432	2.54 (1.79-3.60) *
**Ischaemic stroke**			
Baseline	3,618	5,468	Reference
1st year	338	1,038	1.36 (1.19-1.55) *
2nd year	249	609	1.27 (1.10-1.48) *
3rd year	202	496	1.24 (1.06-1.46) *
4th year	175	370	1.35 (1.14-1.61) *
5th year	153	302	1.49 (1.24-1.79) *
6th year	101	225	1.38 (1.11-1.71) *
≥7 years	153	411	1.17 (0.97-1.41)

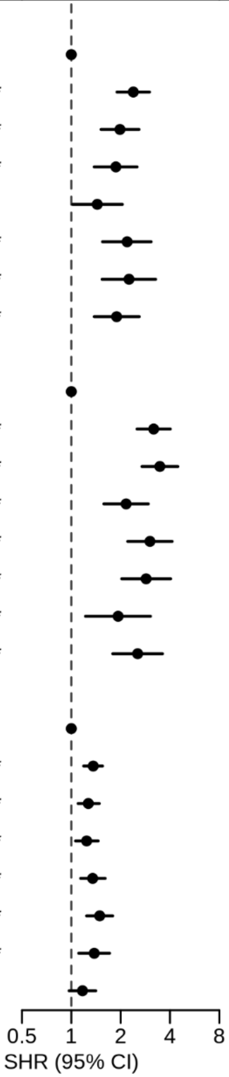

	Cases	Competing events	SHRs (95%CIs)
**Other ischaemic heart disease**			
Baseline	3,898	5,319	Reference
1st year	494	901	2.02 (1.81-2.26) *
2nd year	383	476	2.04 (1.81-2.31) *
3rd year	282	399	1.86 (1.61-2.14) *
4th year	222	303	1.83 (1.57-2.14) *
5th year	175	225	1.89 (1.59-2.24) *
6th year	123	178	1.85 (1.52-2.25) *
≥7 years	201	298	1.75 (1.48-2.07) *
**Pulmonary heart disease**			
Baseline	1,184	5,730	Reference
1st year	390	835	5.38 (4.66-6.22) *
2nd year	217	486	4.06 (3.41-4.83) *
3rd year	169	358	3.99 (3.29-4.84) *
4th year	128	257	4.07 (3.27-5.05) *
5th year	114	222	4.85 (3.85-6.12) *
6th year	63	166	3.69 (2.74-4.98) *
≥7 years	125	279	5.05 (3.99-6.38) *
**Intracerebral haemorrhage**			
Baseline	1,228	5,605	Reference
1st year	97	1,136	0.95 (0.75-1.21)
2nd year	80	677	0.97 (0.75-1.26)
3rd year	63	543	0.91 (0.69-1.22)
4th year	57	414	1.03 (0.75-1.40)
5th year	44	351	1.00 (0.71-1.42)
6th year	27	263	0.84 (0.55-1.26)
≥7 years	52	491	0.92 (0.67-1.28)

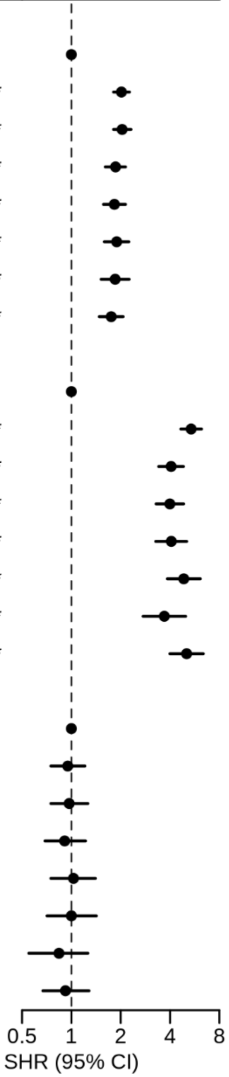

Figure S4. Subdistribution hazard ratios and 95% confidence intervals for long-term CVD risk after the first ECOPD hospitalization

CI, confidence interval; CVD, cardiovascular disease; ECOPD, exacerbation of chronic obstructive pulmonary disease (COPD); SHR, subdistribution hazard ratio.

The SHRs (95% CIs) with asterisks were statistically significant after Bonferroni correction (P<0.05/6).

Models were adjusted for the same covariates as in Table 2.

	Screen-detected		Self-reported		Newly documented		
	Cases	HRs (95%CI)	Cases	HRs (95%CI)	Cases	HRs (95%CI)	P_{int}
**Acute myocardial infarction**							**0.313**
Baseline	501	Reference	199	Reference	28	Reference	
1-2 years	38	1.63 (1.09-2.43)	37	3.73 (2.38-5.83) *	130	1.66 (0.96-2.90)	
3-4 years	28	1.46 (0.91-2.35)	19	2.41 (1.36-4.27) *	58	1.10 (0.61-1.98)	
5-6 years	19	1.61 (0.94-2.78)	18	3.31 (1.80-6.11) *	49	1.60 (0.87-2.95)	
≥7 years	15	1.40 (0.75-2.60)	20	3.63 (1.94-6.82) *	26	1.11 (0.57-2.16)	
**Other ischaemic heart disease**							**<0.001 ***
Baseline	2,372	Reference	1,409	Reference	117	Reference	
1-2 years	169	2.22 (1.84-2.68) *	121	1.33 (1.07-1.66)	587	1.73 (1.33-2.25) *	
3-4 years	100	1.84 (1.45-2.32) *	115	1.74 (1.38-2.20) *	289	1.41 (1.07-1.87)	
5-6 years	57	1.77 (1.31-2.38) *	71	1.63 (1.23-2.17) *	170	1.54 (1.14-2.07)	
≥7 years	54	2.32 (1.71-3.16) *	46	1.26 (0.89-1.77)	101	1.36 (0.97-1.89)	
**Heart failure**							**0.152**
Baseline	349	Reference	203	Reference	38	Reference	
1-2 years	64	3.87 (2.70-5.56) *	51	3.41 (2.30-5.04) *	127	1.49 (0.93-2.38)	
3-4 years	25	1.99 (1.21-3.26)	29	2.56 (1.56-4.20) *	69	1.20 (0.73-2.00)	
5-6 years	13	1.67 (0.88-3.17)	19	2.66 (1.48-4.78) *	39	1.32 (0.75-2.30)	
≥7 years	12	2.29 (1.18-4.42)	11	1.70 (0.81-3.56)	29	1.49 (0.81-2.73)	
**Pulmonary heart disease**							**<0.001 ***
Baseline	529	Reference	597	Reference	58	Reference	
1-2 years	170	5.73 (4.58-7.19) *	225	3.88 (3.20-4.69) *	212	2.46 (1.69-3.58) *	
3-4 years	95	5.15 (3.88-6.82) *	133	3.71 (2.92-4.72) *	69	1.37 (0.89-2.11)	
5-6 years	43	4.47 (3.07-6.51) *	75	3.91 (2.88-5.30) *	59	2.13 (1.34-3.38) *	
≥7 years	37	6.29 (4.18-9.47) *	48	3.70 (2.55-5.35) *	40	2.61 (1.57-4.35) *	
**Ischaemic stroke**							**0.937**
Baseline	2,354	Reference	1,159	Reference	105	Reference	
1-2 years	105	1.37 (1.09-1.72)	80	1.21 (0.93-1.58)	402	1.29 (0.98-1.70)	
3-4 years	66	1.12 (0.85-1.48)	65	1.22 (0.90-1.64)	246	1.23 (0.92-1.65)	
5-6 years	42	1.17 (0.83-1.65)	46	1.23 (0.87-1.73)	166	1.48 (1.09-2.02)	
≥7 years	29	1.09 (0.73-1.62)	36	1.09 (0.74-1.59)	88	1.13 (0.80-1.59)	
**Intracerebral haemorrhage**							**0.066**
Baseline	859	Reference	329	Reference	40	Reference	
1-2 years	33	0.81 (0.55-1.21)	28	0.84 (0.53-1.33)	116	1.05 (0.67-1.67)	
3-4 years	24	0.76 (0.48-1.21)	20	0.81 (0.47-1.38)	76	1.02 (0.63-1.66)	
5-6 years	20	0.99 (0.59-1.65)	24	1.35 (0.80-2.29)	27	0.58 (0.33-1.04)	
≥7 years	13	0.85 (0.46-1.58)	17	1.05 (0.57-1.92)	22	0.74 (0.40-1.36)	

0.5 1 2 4 8 16 HR (95% CI)

Figure S5. Associations between first ECOPD hospitalization and long-term CVD risk stratified by different types of COPD patients

CI, confidence interval; COPD, chronic obstructive pulmonary disease; CVD, cardiovascular disease; ECOPD, exacerbation of COPD; HR, hazard ratio.
The HRs (95% CIs) with asterisks were statistically significant after Bonferroni correction (P<0.05/18), so were the P_{int} (<0.05/6).
The multivariable models were adjusted for the same covariates as in Table 2.
